# Temporal and habitat adaptations in *Drosophila subobscura* populations: changes in chromosomal inversions

**DOI:** 10.1007/s10709-025-00232-9

**Published:** 2025-04-25

**Authors:** Goran Zivanovic, Concepció Arenas, Francesc Mestres

**Affiliations:** 1 Department of Evolutionary Biology, Institute for Biological Research “Sinisa Stankovic” - National Institute of Republic of Serbia, University of Belgrade, Bulevar Despota Stefana 142, Belgrade, 11000 Serbia; 2 Departament de Genètica, Microbiologia i Estadística, Secció d’Estadística, Universitat de Barcelona, Av. Diagonal 643, Barcelona, 08028 Spain; 3 Departament de Genètica, Microbiologia i Estadística, Secció de Genètica Biomèdica, Evolutiva i Desenvolupament - IRBio (Institut de Recerca per la Biodiversitat), Universitat de Barcelona, Av. Diagonal 643, Barcelona, 08028 Spain

**Keywords:** *Drosophila subobscura*, Chromosomal inversions, Environment, Global warming, Natural selection, Adaptation

## Abstract

In insects, chromosomal inversion polymorphism has been related with different adaptations, including global warming. Regarding this environmental change, *Drosophila subobscura* stands out as a useful model species due to its rich inversion polymorphism covering the whole karyotype. The main aims of this research were to analyze the differentiation of this polymorphism in Jastrebac Mt. (Serbia) depending on the different habitats (beech and oak forests) and over time. These latter changes were studied in relation to climatic variables (mean, minimum and maximum temperatures, humidity and rainfall). Microdifferentiation was observed between beech and oak forests, mainly for the A and O chromosomes. However, the changes over time turned out to be larger than those due to habitat. In Jastrebac Mt., temperatures increased over time, with this increase being significant for mean and minimum one. The Multidimensional Scaling analysis showed a relation between chromosomal inversions and temperatures (mainly minimum) in Jastrebac Mt. and other Serbian populations of *D. subobscura*. In beech forest of Jastrebac Mt., the Chromosomal Thermal Index increased over time from 1990 to 1994, but showing a possible stabilization in 2023. This result was observed in other studied Serbian populations. Although those are preliminary results, it might hypothesize that there may be a threshold for the action of natural selection, increasing ‘warm’ adaptive inversions and decreasing ‘cold’ ones. The possible reasons for this hypothesis are also discussed.

**Supplementary Information:**

The online version contains supplementary material available at 10.1007/s10709-025-00232-9.

## Introduction

A classic cornerstone in Evolution is to understand how natural selection chose among the available genetic variability which genes confer the adaptation of organisms to certain environmental conditions (Singh and Singh 2018; Barton 2022). To ascertain which genes are the target of natural selection is a relevant question, although it is not easy to answer. In this context, environmental changes due to global warming are an excellent opportunity to study genetic adaptation of species due to natural selection. There is a large number of examples of this kind of adaptation (for instance, Horie 2019; Kosaka et al. 2019; Verheyen and Stoks 2019; Lee et al. 2020; Matz et al. 2020; Fernández et al. 2022). One of the pioneers in studying the genetic basis of adaptations was Dobzhansky, using chromosomal inversions of *Drosophila pseudoobscura* (Dobzhansky 1970; Lewontin et al. 1981). Although many species of genus *Drosophila* also present inversions, *Drosophila subobscura* has demonstrated to be a valuable model species, because its rich inversion chromosomal polymorphism for its whole karyotype is adaptive to climatic change (see Rodríguez-Trelles and Rodríguez 1998; Balanyà et al. 2006, 2009; Rodríguez-Trelles et al. 2013; Zivanovic et al. 2023 and references therein). In this species, ‘warm’ and ‘cold’ adapted inversions were defined by different authors based on multivariate analyses using inversion polymorphism and climatic data, and also by the latitudinal clinal distribution of the frequencies of certain inversions (Menozzi and Krimbas 1992; Balanyà et al. 2006; Rego et al. 2010; Arenas et al. 2024). In *D. subobscura*, changes in composition and frequencies of chromosomal inversions or their combinations, the so-called arrangements, have been observed over time. With the aim of simplifying, ‘inversions’ are used throughout the text to refer to both, individual inversions and arrangements. Thus, ‘warm’ adapted inversions were recently found for the first time in populations located in rather high latitudes, whereas other ‘warm’ adapted inversions increased in frequency. On the contrary, ‘cold’ adapted inversions generally decreased in frequency in species populations. These qualitative and quantitative changes in inversion composition were observed in *D. subobscura* from its central area of distribution (e.g., Orengo and Prevosti 1996; Rodríguez-Trelles and Rodríguez 1998; Solé et al. 2002; Balanyà et al. 2004; Zivanovic and Mestres 2011; Rodríguez-Trelles et al. 2013; Zivanovic et al. 2015, 2023; Orengo et al. 2016; Galludo et al. 2018), in marginal populations of the species, as Iran (Khadem et al. 2022), in the mid-Atlantic isolated island of Madeira (Madrenas et al. 2020) and also in American colonizing populations (Balanyà et al. 2006, 2009; Arenas et al. 2024). However, in the genus *Drosophila*, inversions cannot only be adaptive directly to temperature, but to other aspects related in some way to temperature. Furthermore, they can also adapt to other environmental conditions present in ecosystems (Sperlich and Pfriem 1986; Krimbas 1992, 1993; Hoffmann et al. 2004; Santos et al. 2004; Hoffmann and Rieseberg 2008; Galludo et al. 2018; Kapun and Flatt 2019).

Furthermore, in *Drosophila subobscura*, microdifferentiation regarding the inversion chromosomal polymorphism was reported by Kimbas and Alevizos (1973). In this case, flies trapped 50 m apart in Mt. Parnes (Greece) showed differences in inversion frequencies. In Jastrebac Mt. (Serbia), Zivanovic et al. (1995) found differences for the inversion frequencies of A, J, U and E chromosomes between samples from beech and oak forests, separated about 3.5 km apart. However, in mountain Goč (Serbia), no significant differences in the overall frequency distribution of inversions were observed between *D. subobscura* populations from beech and oak forests, separated by about 4 km (Rasic et al. 2008). However, these authors reported particular differences for several E and O inversions.

From all these observations, it is possible to hypothesize that the chromosomal inversion polymorphism of *D. subobscura* could vary over time, but in different ways depending on the ecological conditions of the habitat, even if the capture sites were located quite close to each other. For instance, it is known that different kind of forests can better withstand the drought effects caused by climatic change. For this reason, it is expected that oak forest would better adapt than beech forest (Rubio-Cuadrado et al. 2018; Gazol et al. 2018; Kasper et al. 2022). To analyze this hypothesis, our main aim was to collect again flies in Jastrebac Mt. (2023) exactly in the same month (June) and place, both in beech and oak forests, to obtain their chromosomal polymorphism and to compare the results with those recorded in 1990. As a second aim, we studied the evolution of this polymorphism in the beech forest in a temporal series (1990, 1993, 1994 and 2023) to analyze the possible effects of climatic change on chromosomal inversion composition. Finally, an attempt was made to study the possible effect that some meteorological variables could produce by stimulating adaptive changes on chromosomal inversion polymorphism. The changes over time of this polymorphism are still considered a fundamental issue in the evolution of species.

## Materials and methods

### *D. subobscura* samples and chromosomal inversion analysis

Flies were collected at the Jastrebac Mt. (central Serbia) in two different habitats located approximately 3.5 km apart, a beech forest (43º 24’ 28.6” N and 21º 22’ 56.6” E; ~500 m a.s.l.) and an oak forest (43º 28’ 26” N and 21º 21’ 01” E; ~350 m a.s.l.). There were exactly the same places of 1990, 1993 and 1994 *D. subobscura* samples (Zivanovic et al. 1995). Both places differ regarding biota, microclimate and soil composition. In Jastrebac Mt. beech forest, the absolutely dominant tree is *Fagus moesiaca*. Several other tree species are also present, but very rare like *Carpinus betulus*, *Acer heldreichi*, *Acer platanoides*, *Tilia grandifolia*, *Acer pseudoplatanus*. Brushwood plants are represented by *Sambucus nigra*, *Evonimus latifolie*, *Festuca drymeia*, *Carex pilosa*, and others. On the other hand, the oak forest is composed dominantly by *Quercus cerris* together with other tree species like *Carpinus orientalis*, *Acer monspessulanum*, *Crataegus lacinata*, *Fraxinus ornus*, *Juniperus communis*. Its brushwood plants are represented by *Fragaria vesca*, *Primula vulgaris*, *Veronica officinalis*, *Aristolochia pallida*, *Helleborus odorus*, and others. For all these aspects, both locations are considered different habitats (Horvat 1963), where habitat is defined as a local environment (Lincoln et al. 1992).

Sampling procedure was equivalent in both places: 40 plastic boxes filled with fermented apples by *Saccharomyces cerevisiae* were used as baits. *D. subobscura* flies were netted during 5 h (3 p.m. − 8 p.m.). Beech and oak collections were obtained on 12th − 13th and 14th June 2023, respectively. The collecting process was interrupted several times by heavy rain, although enough large sample sizes were obtained in both places. Once in the laboratory, wild males and females were classified, and individual isofemale lines were founded with collected females. Wild males and sons of wild females were individually crossed in vials containing virgin females from the Kussnacht strain. This strain is used as a chromosomal reference, because it is homokaryotypic for the standard arrangements in all acrocentric chromosomes (A, E, J, U and O). The chromosomal slides were obtained by dissecting third instar larvae from the F_1_, and polytene chromosomes were stained and squashed in aceto-orcein solution. The chromosomal inversions identification was carried out using the chromosomal maps described by Kunze-Mühl and Müller (1958) and Krimbas (1992, 1993). At least eight larvae were analyzed from the progeny of each cross, to obtain the karyotypes with a probability higher than 0.99. The conditions for all crosses were: 18ºC, 60% relative humidity and 12 h/12 h light/dark cycle. Finally, according to the criterions developed by Menozzi and Krimbas (1992), Rego et al. (2010) and Arenas et al. (2018), chromosomal inversions were thermally classified as follow: ‘C’ (‘cold’ adapted), ‘W’ (‘warm’ adapted) and ‘N’ (‘non-thermal’ adapted).

### Meteorological data and statistical procedures

The meteorological data from Jasterbac were obtained from The Serbian Republic Hydrometeorological Service: mean temperature (Tmean), maximum temperature (Tmax), minimum temperature (Tmin), humidity and rainfall. The units used for temperature, humidity and rainfall were centigrade degrees, percentage and millimeters of precipitation, respectively. Unfortunately, several meteorological data of Jastrebac Mt. were not available (no information of climatic data for 1990, humidity for 1993 and Tmax for 2011). Particular climatic conditions of beech and oak forests were not available.

Fisher’s exact test was used (statistically significant *P* < 0.05) to compare the chromosomal inversion composition between different places and years, and to study the departure of the observed frequencies of chromosomal karyotypes from Hardy-Weinberg expectations. A bootstrap procedure (100,000 runs) was used to obtain the corresponding *P* values. The FDR correction (Benjamini and Hochberg 1995) was applied whenever there were multiple comparisons, and it was reported as significant for *P* < 0.05. The index of free recombination (*IFR*) was also calculated (Carson 1955). All statistical computations were carried out by means of *basic* and *vegan* packages of R language (R Development Core Team 2014). Moreover, comparisons between the samples from beech forest (1990, 1993, 1994 and 2023) and oak forest (1990 and 2023) populations were carried out using all *D. subobscura* chromosomes (A, E, J, U and O). With these six populations and using the Bhattacharyya distance (Bhattacharyya 1946), a PCoA (Principal Coordinate Analysis) was performed according to Balanyà et al. (2006) and Mestres et al. (2009). Furthermore, with the same data, a GEVA-Ward cluster was computed, due to it is considered an appropriate procedure for chromosomal inversion data (Irigoien et al. 2010; Zivanovic et al. 2016). Finally, the Pearson cophenetic correlation was calculated to quantify how accurately the cluster preserved the pairwise distances between the original populations. Also, multivariate analyses were computed with the six Jastrebac Mt. and other Balkan populations (Zivanovic et al. 2023). Samples from Mt. Parnes (Greece) (Araúz et al. 2009) and Font Groga (Barcelona, Spain) (Galludo et al. 2018) populations were also included in the analyses as outgroups. In this sense, they are useful, because their inversion chromosomal polymorphism is rather different that those from Serbian populations. In this case, only the O chromosome, the most polymorphic one, was used. As in the previous analyses, a PCoA and a GEVA-Ward cluster were computed.

Variations of climatic variables (Tmean, Tmax, Tmin, humidity and rainfall) over time (1991–2023) were studied with temporal series. On the other hand, in all Jastrebac Mt. populations, chromosomal thermal adaptation was measured by computing *CTI* (Chromosomal Thermal Index), described in Arenas et al. (2018). This index is developed in the following way: let W, C and TA be the total number of ‘warm’, ‘cold’, and thermally adapted chromosomes (TA = W+C). The chromosomal thermal index is defined by:

$$\:CTI=\frac{W-C}{W+C}=\frac{W-C}{TA}.$$

**Fig. 1:**
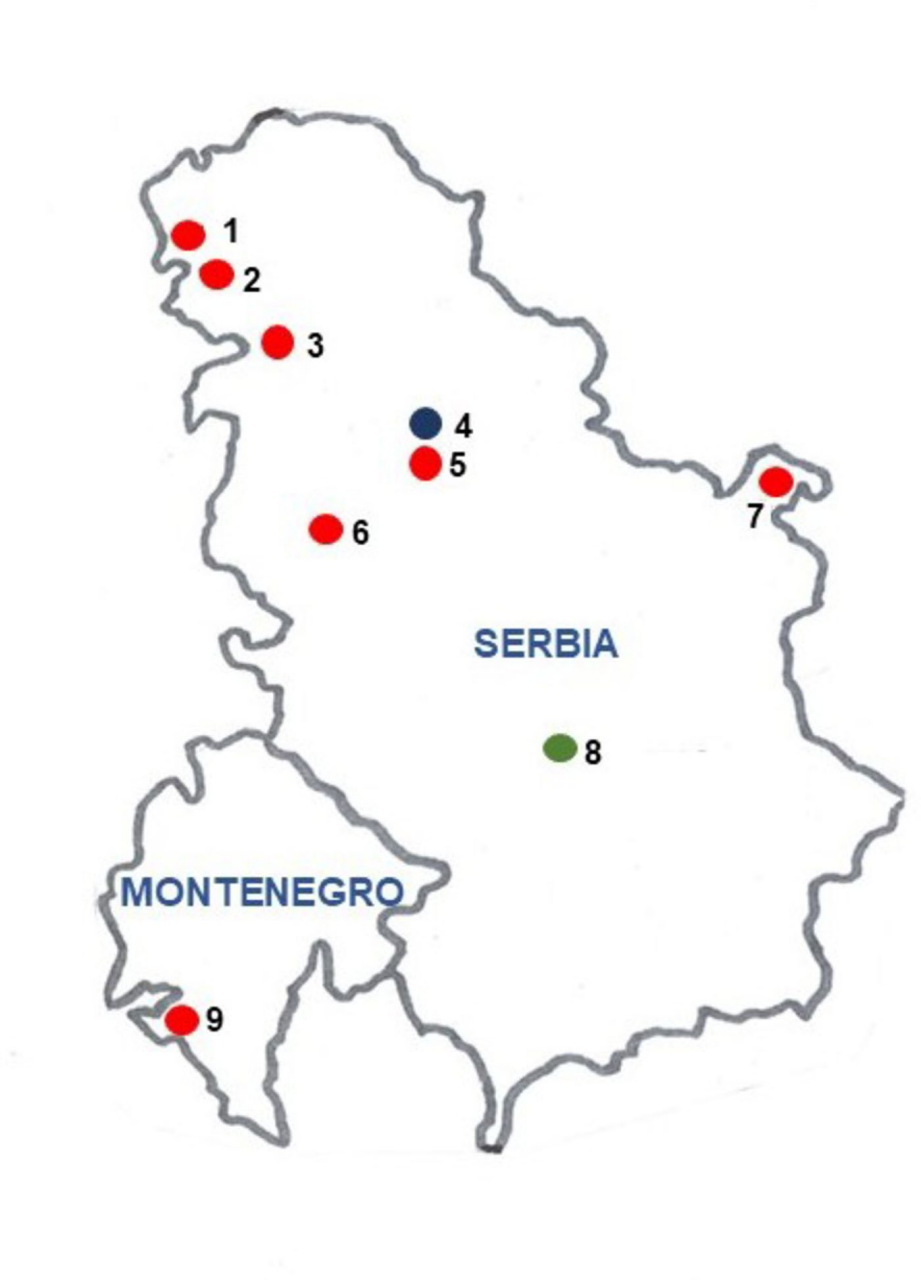
Map showing the location of theSerbian and Montenegrin *D. subobscura* populations used. Numbers indicate the following populations: (1) Apatin; (2) Kamariste; (3) Fruska Gora; (4) Belgrade (in dark blue; shown as geographical reference); (5) Avala; (6) Petnica; (7) Djerdap; (8) Jastrebac (in green); (9) Zanjic

This index allows to quantify the thermal adaptation of a population according to its composition of ‘warm’ and ‘cold’ adapted inversions. It takes values in [-1,1] and measures an excess of ‘warm’ or ‘cold’ chromosomes (positive or negative sign, respectively). It is easy to check that $\:CTI=2p-1$, where $\:p=\frac{W}{TA}$ is the probability, for an adapted chromosome, of been a ‘warm’ chromosome. In order to know if *CTI* matches a certain value or to compare *CTI* values between populations, exact and asymptotic confidence intervals and hypothesis test were constructed. All comparisons among these values were studied using this statistical test also described in Arenas et al. (2018). Finally, a MDS (Multidimensional Scaling) analysis was computed for several Serbian populations in which the following climatic data were available for the month of June: Tmean, Tmax, Tmin, humidity and rainfall. Specifically, these populations were: Jastrebac Mt., beech forest in 1994 (Zivanovic et al. 1995) and 2023 (present research); Apatin,1994 and 2018 (Zivanovic et al. 2019); Petnica, 1995 (Zivanovic et al. 2012 and 2019-2022 (Zivanovic et al. 2023), Kamariste, 1996 (Zivanovic et al. 2002), Djerdap, 2001 and 2002 (Zivanovic 2007) and Avala, 2004 and 2011 (Zivanovic et al. 2015) and 2014–2017 (Zivanovic et al. 2021). The geographical location of these populations is presented in Fig. 1. In the analysis, the whole chromosomal polymorphism for inversions was used.      

## Results

### The chromosomal inversion polymorphism from Jastrebac Mt. (beech and oak forests)

The chromosomal inversion polymorphism of *D. subobscura* for the beech and oak forests from Jastrebac Mt. (June 2023) is presented in Table 1. In the case of beech forest, U_1+8+2_, E_1+2+9+12_ (both ‘warm’ adapted), O_3+4+6_, O_3+4+17_ and O_3+4+22_ inversions were detected for the first time in 2023 (Supplementary Table S1). Regarding oak forest, U_1+8+2_, E_1+2+9+12_, O_3+4+8_ (all three ‘warm’ adapted), J_3+4_, U_1+2+3_, O_3+4+6_, O_3+4+7_ and O_3+4+22_ inversions appeared in 2023 for the first time (Supplementary Table S2). Furthermore, O_3+4+2_, an inversion previously reported in Jastrebac Mt., was absent in 2023 samples. Infrequent inversions, as U_1_, O_6_ and O_15_, are not considered in this comparison. When comparing beech and oak samples from 2023 (Table 2), only A and O chromosomes showed significant differences (*P* = 0.0020, adjusted *P* = 0.0050 and *P* = 0.0060, adjusted *P* = 0.0125, respectively). No significant differences were observed for the other chromosomes: *P* = 1.0, adjusted *P* = 1.0 for the J chromosome; *P* = 0.7103, adjusted *P* = 0.8072 for the U chromosome and *P* = 0.2517, adjusted *P* = 0.2996 for the E chromosome.

**Table 1:** Frequencies of *D. subobscura* chromosomal arrangements of Jastrebac Mt. (June 2023), from Beech and oak

Chrom. arrangement	Thermal adapt.	Beech		Oak	
		n	%	n	%
A_st_	C	27	33.7	19	27.1
A_1_	C	26	32.5	42	60.0
A_2_	W	27	33.7	9	12.8
Total		80		70	
J_st_	C	35	21.8	31	22.1
J_1_	W	123	76.8	107	76.4
J_3+4_	N	2	1.2	2	1.4
Total		160		140	
U_st_	C	11	6.8	16	11.4
U_1_	N	2	1.2	2	1.4
U_1+2_	W	84	52.5	74	52.8
U_1+2+3_	N	3	1.8	3	2.1
U_1+2+6_	N	52	32.5	41	29.3
U_1+8+2_	W	8	5.0	4	2.8
Total		160		140	
E_st_	C	46	28.7	48	34.3
E_8_	N	35	21.8	30	21.4
E_1+2_	N	8	5.0	6	4.3
E_1+2+9_	W	64	40.0	43	30.7
E_1+2+9+12_	W	7	4.3	13	9.3
Total		160		140	
O_st_	C	27	16.8	33	23.6
O_6_	N	0	0	1	0.7
O_3+4_	W	90	56.2	55	39.3
O_3+4+1_	W	24	15.0	14	10.0
O_3+4+6_	N	5	3.1	4	2.8
O_3+4+7_	N	3	1.8	4	2.8
O_3+4+8_	W	4	2.5	11	7.8
O_3+4+17_	N	1	0.6	0	0
O_3+4+22_	N	6	3.7	18	12.8
Total		160		140	
*CTI*		0.430		0.272	

n = number of chromosomes. With regard to thermal adaptation, ‘C’, ‘W’ and ‘N’ stand for ‘cold’, ‘warm’ and ‘non-thermal’ adapted, respectively. This classification was according to Menozzi and Krimbas (1992) and Rego et al. (2010)

**Table 2:** Inversion chromosomal polymorphism comparison between 1990 and 2023 for the Beech and oak forest samples from Jastrebac Mt. For each comparison, the corresponding *P* value and the adjusted *P* value (in brackets) are presented

Chromosome A		
	Beech 2023	Oak 2023
Beech 1990	**0.0010** **(0.0031)**	**0.0010** **(0.0031)**
Oak 1990	**0.0160** **(0.0250)**	**0.0010** **(0.0031)**
Oak 2023	**0.0020** **(0.0050)**	

Chromosome J		
	Beech 2023	Oak 2023
Beech 1990	0.9481 (0.9876)	0.9451 (0.9876)
Oak 1990	0.1638 (0.2289)	0.1918 (0.2435)
Oak 2023	1.0000 (1.0000)	

Chromosome U		
	Beech 2023	Oak 2023
Beech 1990	**0.0010** **(0.0031)**	**0.0140** **(0.0250)**
Oak 1990	**0.0010** **(0.0031)**	**0.0020** **(0.0050)**
Oak 2023	0.7103 (0.8072)	

Chromosome E		
	Beech 2023	Oak 2023
Beech 1990	0.1648 (0.2289)	**0.0160** **(0.0250)**
Oak 1990	0.1948 (0.2435)	**0.0010** **(0.0031)**
Oak 2023	0.2517 (0.2996)	

Chromosome O		
	Beech 2023	Oak 2023
Beech 1990	**0.0030** **(0.0068)**	**0.0010** **(0.0031)**
Oak 1990	**0.0080** **(0.0154)**	**0.0010** **(0.0031)**
Oak 2023	**0.0060** **(0.0125)**	

Significant values appear in bold

The karyotypes for both samples of Jastrebac Mt. (beech and oak forests) are shown in Table 3. Deviations from Hardy Weinberg equilibrium were not observed in either beech (*P* = 1 for the whole karyotype; *P* = 1, *P* = 0.985, *P* = 0.998 and *P* = 0.970 for the J, U, E and O chromosomes, respectively) or oak populations (*P* = 1 for the whole karyotype; *P* = 0.889, *P* = 0.816, *P* = 0.993 and *P* = 0.999 for the J, U, E and O chromosomes, respectively). In the beech and oak forest samples of 2023, the *IFR* values (Table 3) were very similar (82.27 ± 0.78 and 82.37 ± 1.03) and in accordance with estimates from populations situated in the central area of *D. subobscura* distribution (Krimbas 1992).

**Table 3:** Frequencies of chromosomal karyotypes of *D. subobscura* from Jastrebac Mt. (June 2023), from beech and oak

Karyotypes	Beech		Oak	
	n	%	n	%
J_st_/J_st_	4	5.0	5	7.1
J_st_/J_1_	27	33.7	21	30.0
J_1_/J_1_	47	58.7	42	60.0
J_1_/J_3+4_	2	2.5	2	2.8
Total	80		70	
U_st_/U_st_	0	0	2	2.8
U_st_/U_1+2_	9	11.2	11	15.7
U_st_/U_1+2+6_	1	1.2	1	1.4
U_st_/U_1+8+2_	1	1.2	0	0
U_1_/U_1+2_	1	1.2	2	2.8
U_1_/U_1+2+6_	1	1.2	0	0
U_1+2_/U_1+2_	22	27.5	19	27.1
U_1+2_/U_1+2+3_	1	1.2	2	2.8
U_1+2_/U_1+2+6_	25	31.2	21	30.0
U_1+2_/U_1+8+2_	4	5.0	0	0
U_1+2+3_/U_1+2+6_	2	2.5	1	1.4
U_1+2+6_/U_1+2+6_	10	12.5	8	11.4
U_1+2+6_/U_1+8+2_	3	3.7	2	2.8
U_1+8+2_/U_1+8+2_	0	0	1	1.4
Total	80		70	
E_st_/E_st_	5	6.2	11	15.7
E_st_/E_1+2_	4	5.0	2	2.8
E_st_/E_1+2+9_	21	26.2	14	20.0
E_st_/E_1+2+9+12_	2	2.5	1	1.4
E_st_/E_8_	9	11.2	9	12.9
E_1+2_/E_1+2+9_	3	3.7	2	2.8
E_1+2_/E_1+2+9+12_	0	0	1	1.4
E_1+2_/E_8_	1	1.2	1	1.4
E_1+2+9_/E_1+2+9_	13	16.2	8	11.4
E_1+2+9/_E_1+2+9+12_	2	2.5	3	4.3
E_1+2+9_/E_8_	12	15.0	8	11.4
E_1+2+9+12_/E_1+2+9+12_	0	0	3	4.3
E_1+2+9+12_/E_8_	3	3.7	2	2.8
E_8_/E_8_	5	6.2	5	7.1
Total	80		70	
O_st_/O_st_	3	3.7	5	7.1
O_st_/O_3+4_	17	21.2	11	15.7
O_st_/O_3+4+1_	4	5.0	4	5.7
O_st_/O_3+4+6_	0	0	1	1.4
O_st_/O_3+4+7_	0	0	2	2.8
O_st_/O_3+4+8_	0	0	4	5.7
O_st_/O_3+4+22_	0	0	1	1.4
O_6_/O_3+4+1_	0	0	1	1.4
O_3+4_/O_3+4_	23	28.7	10	14.3
O_3+4_/O_3+4+1_	14	17.5	5	7.1
O_3+4_/O_3+4+6_	1	1.2	2	2.8
O_3+4_/O_3+4+7_	3	3.7	1	1.4
O_3+4_/O_3+4+8_	4	5.0	6	8.6
O_3+4_/O_3+4+17_	1	1.2	0	0
O_3+4_/O_3+4+22_	4	5.0	10	14.3
O_3+4+1_/O_3+4+1_	1	1.2	0	0
O_3+4+1_/O_3+4+6_	4	5.0	1	1.4
O_3+4+1_/O_3+4+22_	0	0	3	4.3
O_3+4+7_/O_3+4+22_	0	0	1	1.4
O_3+4+8_/O_3+4+22_	0	0	1	1.4
O_3+4+22_/O_3+4+22_	1	1.2	1	1.4
Total	80		70	
*IFR*	82.27 ± 0.78		82.37 ± 1.03	

n = number of karyotypes

### Chromosomal inversion polymorphism changes over time and habitat

The inversion polymorphism was compared for each individual chromosome between samples from 1990 (beech and oak forests) and 2023 (beech and oak forests) (Table 2). For the A, U and O chromosomes, all comparisons were significant; for the E chromosome, only those comparisons involving oak forest 2023 were significant; for the J chromosome no comparison was significant. In particular, significant changes over time (1990–2023) were detected in beech forest for chromosomes A, U and O chromosomes. However, the comparison over time for oak forest proved to be significant for the A, U, E and O. Finally, in 2023, significant changes between beech and oak forests were observed for the A and O chromosomes.

**Fig. 2:**
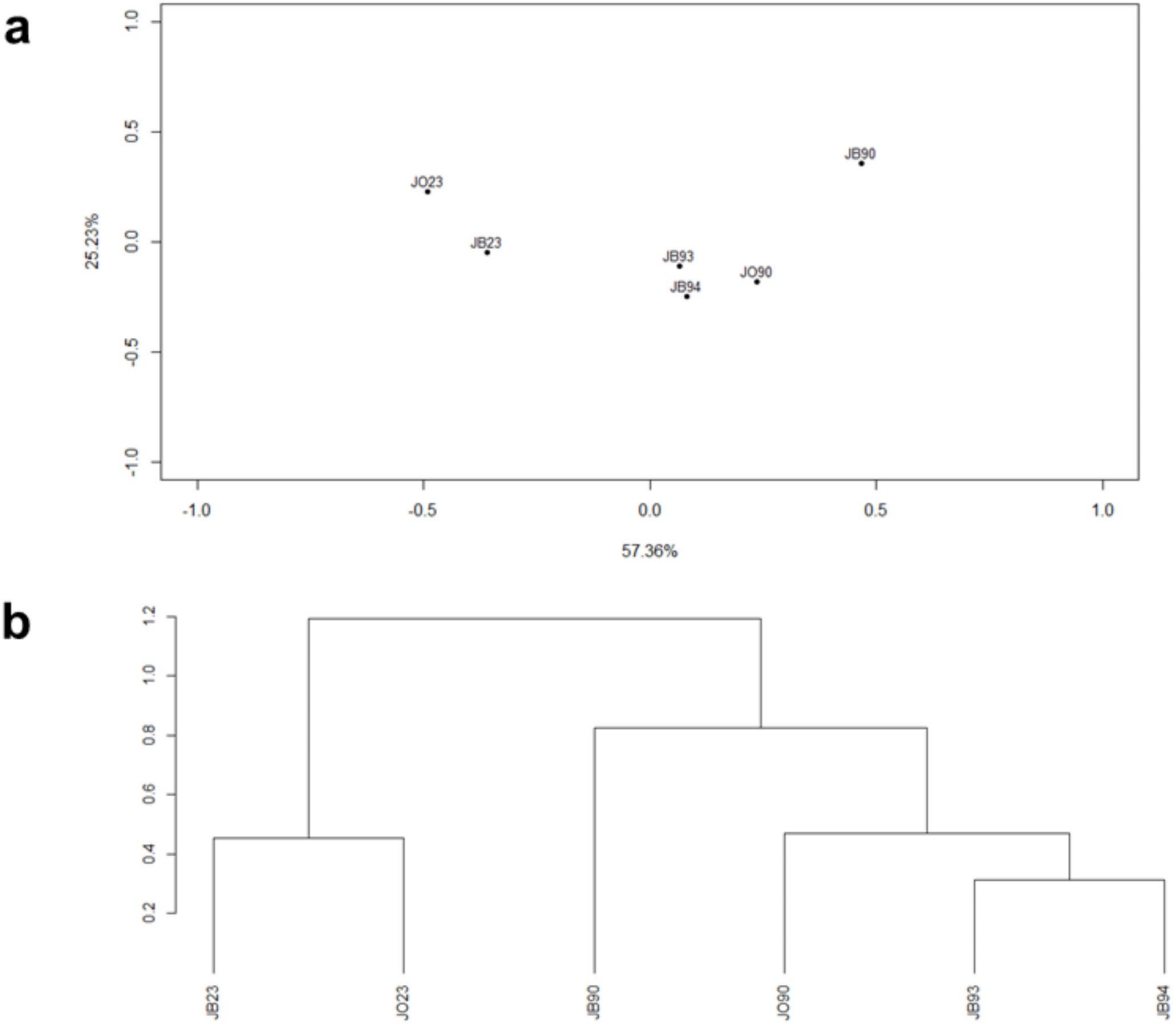
Comparison between the chromosomal inversions between the six Jastrebac Mt. samples using multivariate analysis. **a** Principal Coordinate Analysis. Samples are approximately separated by time, being at the left those from 2023, at the right those from 1990 and centered those from 1993 and 1994. **b** GEVA-Ward cluster. The first partition differentiates both 2023 samples from the others. Abbreviations: JB90 (Jastrebac Mt. beech 1990), JO90 (Jastrebac Mt. 1990 oak), JB93 (Jastrebac Mt. 1993 beech), J94B (Jastrebac Mt.1994 beech), JB23 (Jastrebac Mt. 2023 beech) and JO23 (Jastrebac Mt. 2023 oak)

The chromosomal inversion composition between the six samples available for Jastrebac Mt. (Jastrebac Mt. beech 1990, Jastrebac Mt. 1990 Oak, Jastrebac Mt. 1993 beech, Jastrebac 1994 beech, Jastrebac Mt. 2023 beech and Jastrebac Mt. 2023 oak) was compared and the multivariate analyses (Principal Coordinate Analysis and GEVA-Ward cluster) results are shown in Fig. 2. In the PCoA analysis (Fig. 2a), the first, second and third axis explained 57.36%, 25.23% and 9.50% of the variability, respectively. According to the first axis, samples are separated by time, being those from 2023 at the left, those from 1993 to 1994 centered and those from 1990 at the right. The time variable would be the main differentiating factor for the inversion polymorphism, being more relevant than the habitat variable. Similar results were also observed for the phylogenic tree (Fig. 2b), where the first partition separated recent samples (2023) from the older ones (1990, 1993 and 1994).

Finally, the same kind of multivariate study was carried out, but using the O chromosomal inversions from different Balkan and Font Groga (Barcelona) populations. Regarding the PCoA study (Supplementary Fig. 1), the first, second and third axis explained 36.78%, 18.65% and 10.68% of the variability, respectively. The 2023 Jastrebac Mt. beech and oak forest samples appeared closer than the Jastrebac Mt. samples from previous years. Equivalent results could be observed for the phylogenic tree (Supplementary Fig. 2). Both samples from Jastrebac Mt. (2023) were situated together in one cluster in the second partition of the tree, whereas samples from Jastrebac Mt. collected in previous years appeared in the other group of this partition.

### Climatic change and chromosomal polymorphism

For the period 1991–2023, the variation of studied climatic variables in Jastrebac Mt. is presented in Supplementary Fig. 3. All temperatures increased over time: Tmean (*P* = 0.026, with a slope of 0.050), Tmax (*P* = 0.198, with a slope of 0.038), Tmin (*P* = 0.015, with a slope of 0.039), being this augment significant for Tmean and Tmin. Furthermore, the difference between Tmax and Tmin did not present an increase (*P* = 0.951, with a slope of − 0.001). Finally, humidity did not augment (*P* = 0.697, with a slope of 0.042), whereas rainfall increased over time, but not significantly (*P* = 0.084, with a slope of 2.097). Thus, climatic change was observed in Jastrebac Mt. and according to global warming expectations.

**Fig. 3:**
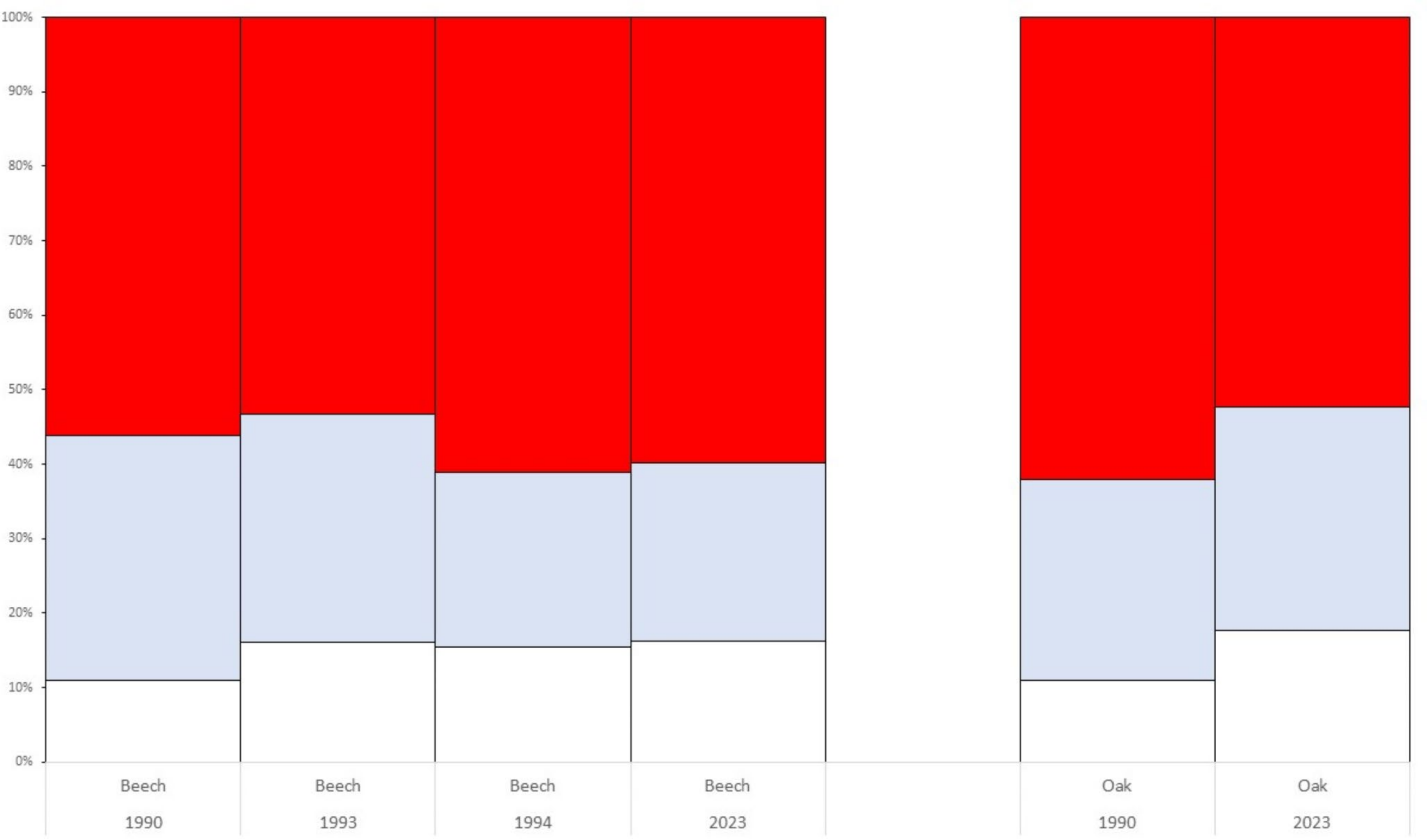
Percentages of ‘warm’ (red), ‘cold’ (blue) and ‘non-thermal’(white) adapted inversions in beech (1990, 1993, 1994 and 2023) and oak (1990 and 2023) from Jastrebac Mt

The *CTI* values for each studied sample from Jastrebac Mt. were: 0.120 (1990 beech), 0.151 (1993 beech), 0.444 (1994 beech), 0.430 (2023 beech), 0.392 (1990 oak) and 0.272 (2023 oak). There was a clear increment for *CTI* values in beech forest from 1990 to 1994, and a subsequent stabilization (2023). However, the results of oak were rather surprising being higher in 1990 than in 2023. The results of the statistical comparisons of *CTI* values between these six Jastrebac Mt. samples is presented in Table 4. The *CTI* values for 1994 and 2023 beech samples were significantly different with regard to those from previous samples (1990 and 1993), but the comparison between 1994 and 2023 was not. For the oak forest, the significant comparisons of *CTI* values were between 2023 oak sample with 1994 and 2023 beech samples. It is interesting that the comparison between 1990 and 2023 oak samples was not significant. Moreover, the changes over time of ‘warm’, ‘cold’ and ‘non-thermal’ adapted inversions in Jastrebac Mt. forests (beech and oak) are presented in Fig. 3 and Supplementary Table S3. There were not differences in the ‘cold’ adapted inversions. For the ‘warm’ adapted inversions there are three significant results, for the A and U chromosomes. In the case of ‘non-thermal’ adapted inversions, there are two and one significant results for the E and O chromosomes, respectively. Finally, the results of the possible relation between the inversion polymorphism and climatic variables using a MDS are presented in Supplementary Fig. 4. Figures represent the 72.41% of the total variability. According to the first axis, the populations are distributed depending on their inversion composition, being those sampled in 2011 or before located at the left of the figure and those more recently sampled (from 2014 onwards) at the right. Regarding temperature, the main effect on chromosomal inversions seems to be due to Tmin, followed by Tmean and finally Tmax. In general, all temperatures present larger effects in recent populations. Humidity is a relevant factor, and its influence is rather general in all populations studied. Finally, rainfall fluctuates in different locations and years, but it seems that Apatin, Avala and Petnica could be considered drier than the others.

**Table 4:** Statistical comparisons between *CTI* values computed from Jastrebac Mt. populations: 1990 Beech and oak, 1993 Beech, 1994 Beech and 2023 Beech and oak

	1990B	1993B	1994B	2023B	1990O	2023O
1990B	‒	-0.140 (0.9525)	-2.855 (**0.0130**)	-2.792 (**0.0130**)	-1.943 (0.0945)	-0.153 (0.9525)
1993B		‒	-3.129 (**0.0130**)	-3.117 (**0.0130**)	-2.075 (0.0814)	-0.028 (0.9774)
1994B			‒	0.026 (0.9525)	0.848 (0.5944)	2.874 (**0.0130**)
2023B				‒	0.648 (0.7500)	2.822 (**0.0150**)
1990O					‒	1.905 (0.0945)
2023O						‒

In the rows, the values of the statistic test are presented and the corresponding adjusted *P* values are shown below in brackets. Nomenclature used: 1990B (1990 beech), 1993B (1993 beech), 1994B (1994 beech), 1990O (1990 oak) and 2023O (2023 oak). Significant values appear in bold

## Discussion

### Adaptation to habitat through chromosomal inversions

A major focus of evolutionary research remains on understanding the genetic basis of adaptations in insects (Danks 2007; Sheikh et al. 2017; Dillon and Lozier 2019; War et al. 2020; McCulloch and Waters 2023) and, currently, with regard to climate change (Hill et al. 2011; Williams 2016; Garnas 2018; González-Tokman et al. 2020; Halsch et al. 2021; Eickermann et al. 2023; Harvey et al. 2023). In some insect species, this adaptation is achieved through chromosomal inversions (for a revision see Hoffmann et al. 2004; Hoffmann and Rieseberg 2008; Sharakhov and Sharakhova 2024), as is the case in *Drosophila subobscura* (Sperlich and Pfriem 1986; Krimbas 1992, 1993). In the present research, habitat and over time changes of the chromosomal inversions of this species were observed. It is well established that habitat selection, even when considering microhabitat preferences, could be an important factor on *Drosophila* populations (Taylor 1978, 1987). In Jastrebac Mt. (2023), significant differences were detected between beech and oak forests for the A and O chromosomal inversions (Table 2). When the same forests were analyzed in 1990, significant differences were observed for the A and U chromosomes (Zivanovic et al. 1995). The natural breeding sites of *D. subobscura* seems to be decaying fruits and vegetation, plant sap, exudates and slime fluxes, and fungi (Krimbas 1993). Regarding the latter, yeasts and mushrooms stand out as a basic nutritional food for larvae and adults. For beech and oak forests, notable differences between these two types of fungi appear (Kowallik et al. 2015; Liti 2015; Kowallik and Greig 2016; Mašínová et al. 2017; Alsammar and Delneri 2020; Langer and Bußkamp 2021; Siddique et al. 2021; Peris et al. 2023). Thus, these differences in fungi composition could be a biological factor which would produce differentiation between both *D. subobscura* populations. Since oak forests appear to be better adapted to a global warming scenario, especially with regard to desiccation (Rubio-Cuadrado et al. 2018; Kasper et al. 2022), it would be interesting to re-analyze the inversion chromosomal polymorphism of both populations in the future. Other authors previously reported genetic microdifferentiation in *D. subobscura* (Krimbas and Alevizos 1973) and *D. melanogaster* (Wallace 1966). They suggested that natural selection, mobility of flies or genetic drift could be evolutionary forces responsible of it.

### Climatic changes and variation in chromosomal inversion frequencies

Although the difference in chromosomal inversions between beech and oak forests appears to have a prominent effect on adaptation, changes on this polymorphism over time probably show that it plays an even greater role in adaptation to more general environmental factors. It is quite possible that inversions change over time to adapt to climate change and its consequences. In this research and considering the same type of forest, changes over time in the chromosomal inversion polymorphism were recorded (Fig. 2, Supplementary Fig. S1 and S2), being even significant for A, U and O chromosomes (Table 2). Also, the distribution of populations in the MDS agrees with changes over time in the inversion polymorphism (Supplementary Fig. S4). The compiled evidence seemed to indicate that most probably the main environmental factor involved is climatic change (Supplementary Fig. S3), as was also reported in previous studies with *D. subobscura* (Menozzi and Krimbas 1992; Orengo and Prevosti 1996; Rodríguez-Trelles and Rodríguez 1998; Balanyà et al. 2006, 2009; Rodríguez-Trelles et al. 2013; Galludo et al. 2018; Zivanovic et al. 2019, 2021, 2023; Rodríguez-Trelles and Tarrío 2024). In Jastrebac Mt., over time changes in temperature for the period 1991–2023 were observed, being Tmean and Tmin significant. Interestingly, the difference between Tmax and Tmin remained rather constant for this period, indicating that both variables increased in a similar way. The MDS results would indicate a relation between chromosomal inversions and rainfall, humidity and temperatures, mainly with Tmin. Similar results were previously reported (Galludo et al. 2018; Zivanovic et al. 2021, 2023).

### Variation of thermal adapted inversions over time

The possible correspondence of this environmental changes with thermal adapted inversion was also analyzed using the *CTI* index. In beech forest, this index increased in the first samples (1990, *CTI* = 0.120; 1993, *CTI* = 0.151; 1994, *CTI* = 0.444) and it seemed that reached a stabilization (*CTI* = 0.430), likely due to a slowdown or even a stop in the accumulation of ‘warm’ adapted inversions (Fig. 3). Additionally, no significant difference was observed between the *CTI* values from 1994 to 2023 (Table 4). This hypothetical stabilization with regard to *CTI* values has been also observed in other temporal series from recent years in different populations. In these series, it is possible to study, for each population, the difference between the maximum *CTI* value (*CTI*max) and the minimum one (*CTI*min) registered. For instance: Font Groga (Barcelona, Spain), years 2011, 2012, 2013, 2014 and 2015, *CTI*min = 0.285, *CTI*max = 0.408, difference between both values 0.123 (Galludo et al. 2018); Avala (Serbia) years 2014, 2015, 2016 and 2017, *CTI*min = 0.262, *CTI*max = 0.383, difference between both values 0.121 (Zivanovic et al. 2021); Petnica (Serbia), years 2019, 2020, 2021 and 2022, *CTI*min = 0.097, *CTI*max = 0.211, difference between both values 0.114 (Zivanovic et al. 2023). In these cases, the difference is small and very similar, in agreement with the stabilization hypothesis. Those are preliminary data, and thus, more studies are needed for demonstrating or refuting this hypothetical stabilization over extended time periods. On the contrary, in the oak forest, an unexpected significant decrease of this index was observed (1990, *CTI* = 0.392; 2023, *CTI* = 0.272), although it was not significant. This could be explained due to bias effect of sampling or some different environmental conditions between both temporal samples. Also in this case, more samples have to be obtained in the future to properly understand these chromosomal changes in Jastrebac Mt. oak forest.

### The complex scenario of thermal adaptation by chromosomal inversions

Additionally, it was possible to compare the frequencies of ‘cold’, ‘warm’ and ‘non-thermal’ adapted inversion for each chromosome in 1990 and 2023 for beech and oak forests (Supplementary Table S3). No significant change was observed for ‘cold’ adapted inversions. With regard to ‘warm’ adapted inversions, significant changes were only observed for the A and U chromosomes, being relevant the case between 1990 and 2023 beech populations. The U chromosome was previously reported as relevant in thermal adaptation (Zivanovic and Mestres 2011; Galludo et al. 2018; Zivanovic et al. 2015, 2019, 2021). For ‘non-thermal’ adapted inversions, three significant differences were detected for the E and O chromosomes, and two of them belonged to oak forest comparisons (one for each chromosome). Although there is global warming, over time frequency changes of ‘non-thermal’ adapted inversions were reported in different *D. subobscura* populations, and could be due to genetic drift or adaptation to other environmental factors (Zivanovic et al. 2019, 2021, 2023). Maybe, the criteria used to classify the inversions in ‘cold’, ‘warm’ and ‘non-thermal’ adapted (Menozzi and Krimbas 1992; Rego et al. 2010; Arenas et al. 2024), although are useful, should be revisited.

Thermal adaptation is likely a complex trait, where a considerable fraction of genes that control it would be located in inversions considered ‘thermal adapted’, but there could be other genes responsible for this adaptation in other regions of the karyotype. Therefore, in the *Drosophila* genus, it is essential to identify the thermal adapted genes and to ascertain their genomic and karyotypic (inside or outside the inversions) locations. Several researcher groups have obtained valuable results in this topic (for instance, Anderson et al. 2003; Hoffmann et al. 2004; Hoffmann and Weeks 2007; Laayouni et al. 2007; Rako et al. 2007; van Heerwaarden and Hoffmann 2007; Dolgova et al. 2010; Calabria et al. 2012; Pegueroles et al. 2013; Simões and Pascual 2018). It is worth noting, that thermal adaptation in American colonizing populations (Balanyà et al. 2006, 2009) is due to different inversions that those described in the Palearctic area of distribution (Arenas et al. 2024), due to a larger founder effect and natural selection acting on available genetic variability (Ayala et al. 1989; Prevosti et al. 1989).

When analyzing the evolution of the chromosomal inversions of *D. subobscura* over large time periods (20 or 30 years), which implies approximately between 100 and 150 generations in natural populations (assuming and average of 5 generations per year, according to Begon (1976) and Mestres et al. (2001), a fast change in composition and frequencies was observed in populations studied (Orengo and Prevosti 1996; Orengo et al. 2016; Solé et al. 2002; Balanyà et al. 2004, 2006, 2009; Zivanovic et al. 2019; Madrenas et al. 2020; Khadem et al. 2022; Arenas et al. 2024; Rodríguez-Trelles and Tarrío 2024). However, the present research could indicate that the natural selection increasing ‘warm’ adapted inversions (and obviously decreasing ‘cold’ adapted ones) maybe has a threshold. This is an interesting hypothesis that deserved more studies in natural populations of *D. subobscura* and other species of *Drosophila* genus presenting chromosomal inversion polymorphism, for instance, *Drosophila buzzatii* (Fernández-Iriarte et al. 1999; Dahlgaard et al. 2001; Soto et al. 2010), D. *mediopunctata* (Ananina et al. 2004; Batista et al. 2012), D. *melanogaster* (Umina et al. 2005; Hangartner et al. 2015; Kapun and Flatt 2019; Nunez et al. 2024), D. *robusta* (Levitan 2003; Levitan and Edges 2005), or *D. pseudoobscura* (for a revision see Schaeffer 2008). With regard to temperature, selection has to operate under a complex scenario which could be defined by three levels of fluctuating conditions: (1) changes depending on the diurnal time (basically, colder at night and warmer during the day), (2) seasonal changes (alternance of cold and warm seasons) and (3) long-term changes due to global warming (involving large periods of time). Furthermore, these three levels show rather unpredictable fluctuations in temperature (for instance see Robinson 2021; Serrano-Notivoli et al. 2022; Zhang et al. 2022; Yin et al. 2024), including an increase of heatwaves (Barriopedro et al. 2023; He et al. 2023; Wedler et al. 2023; Schmidt 2024). With regard to first level, *D. subobscura* flies have to adapt to cyclic diurnal temperature variations. Furthermore, the diurnal cycles are different depending on the second level (seasonal changes). For instance, seasonal changes in the chromosomal inversion polymorphism were reported in *D. subobscura* (Fontdevila et al. 1983; Rodriguez-Trelles et al. 1996; Rodriguez-Trelles 2003; Zivanovic 2007). In *D. subobscura*, the annual number of generations would oscillate between 4–6 (Begon 1976; Mestres et al. 2001), and they are not equally distributed over the year (Krimbas 1993; Argemí et al. 1999), because reproductive cycle depends on temperature, and this varies according to seasonality (Zivanovic et al. 2023). If possible, flies have to genetically adapt to the temperatures corresponding to each season. Finally, the third level is the background of global warming, increasing temperatures in a fluctuant way, and adaptations, under these circumstances, are also needed. For these reasons, although climatic change is a fact, ‘cold’ adapted inversions could be still needed in many *D. subobscura* populations of its distribution area. In Jastrebac Mt. and for June 2023, the proportion of genotypes presenting at least one ‘cold’ adapted inversion for beech and oak forests was 92.5% and 98.57%, respectively. This proportion for other populations was: Avala June 2017, 95.39% (Zivanovic et al. 2021); Apatin June 2018, 96.67% (Zivanovic et al. 2019); Petnica June 2022, 99.34% (Zivanovic et al. 2023). Also, it should be remembered that genes that are involved in adaptations to other environmental factors or to fitness traits could be found within ‘cold’ adapted inversions or outside of them, but very close to their breakpoints, being inherited together. Thus, strong linkage disequilibrium could be expected. As a consequence, selection favoring those genes, could also explain the maintenance those ‘cold’ adapted inversions.

In summary, chromosomal inversions are genetic reorganizations that allow organisms to adapt by means of natural selection. In *D. subobscura*, it has been demonstrated that they have generated adaptations to different biotic and environmental conditions. One of latter is global warming and predictions seem to indicate that will increase in the following years. For this reason, it will be extremely interesting to follow up the evolution of inversion chromosomal polymorphism in natural populations and to observe whether there is a limit to thermal adaptation.

## Electronic supplementary material

Below is the link to the electronic supplementary material.

Supplementary Material 1.

Supplementary Material 2.

Supplementary Material 3.

Supplementary Material 4.

Supplementary Material 5.

Supplementary Material 6.

Supplementary Material 7

Supplementary Material 8

## Data Availability

No datasets were generated or analysed during the current study.
