# Supplementary material for: Temporal and habitat adaptations in Drosophila subobscura populations: changes in chromosomal inversions
Source: Genetica. 2025 Apr 25;153(1):16. doi: 10.1007/s10709-025-00232-9 (PMC12031780; doi:10.1007/s10709-025-00232-9)
Supplement: Supplementary file 1 — Supplementary Material 1. [file 10709_2025_232_MOESM1_ESM.docx]

**Supplementary Figure S1** Principal Coordinate Analysis of Balkan and Front Groga (Barcelona) populations using the O chromosomal inversions.
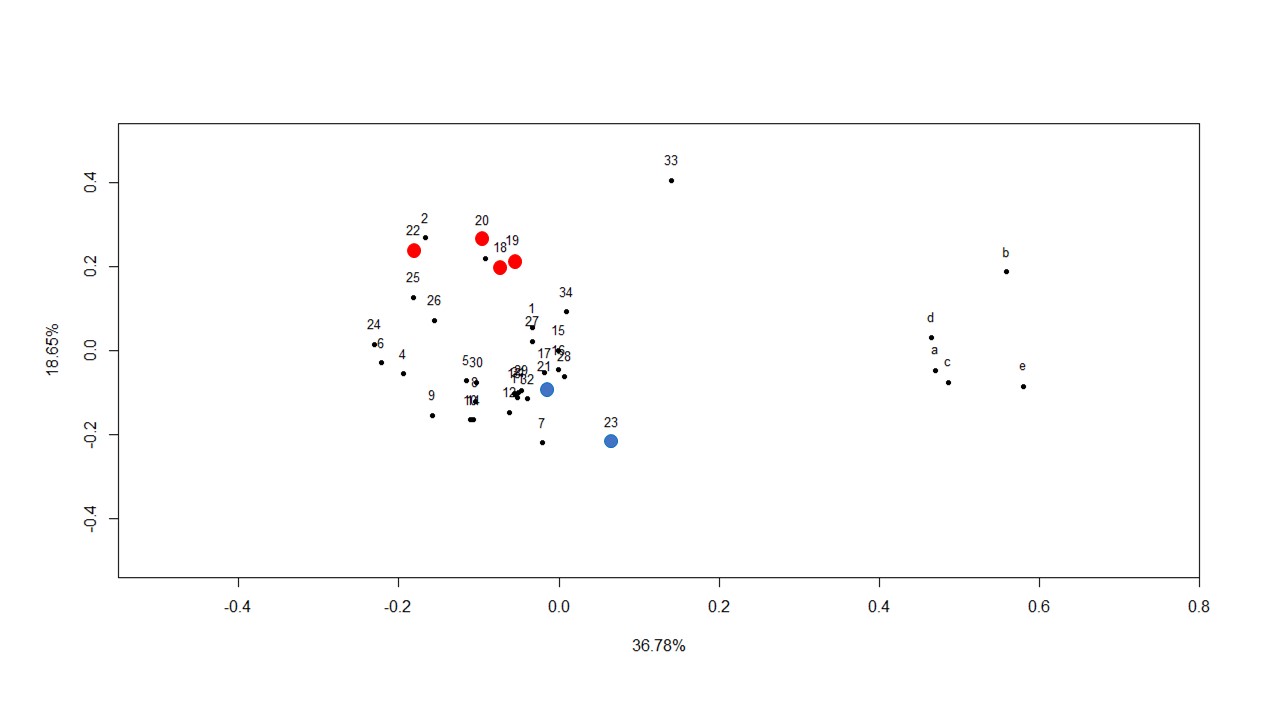


Abbreviations: 1, Mt. Parnes (Greece); 2, Apatin 1994; 3, Apatin 2008; 4, Apatin 2009; 5, Apatin 2018; 6, Avala 2003; 7, Avala 2004 (June); 8, Avala 2004 (Sept.); 9, Avala 2005; 10, Avala 2011; 11, Avala, 2014; 12, Avala 2015; 13, Avala 2016; 14, Avala 2017; 15, Djerdap 2001 (June); 16, Djerdap (August); 17, Djerdap (2002); 18, Jastrebac Mt. (beech) 1990; 19, Jastrebac Mt. (beech) 1993); 20, Jasterbac Mt. (beech) 1994; 21, Jastrebac Mt. (beech) 2023; 22, Jastrebac Mt. (oak) 1990; 23, Jastrebac Mt. (oak) 2023; 24, Kamariste 1996; 25, Petnica (May) 1995; 26, Petnica (June) 1995; 27, Petnica (August) 1995; 28, Petnica 2010; 29, Petnica 2019; 30, Petnica 2020; 31, Petnica 2021; 32, Petnica 2022; 33, Fruska Gora; 34, Zanjic (Montenegro) 1997; a, Font Groga 2011; b, Font Groga 2012; c, Font Groga 2013; d, Font Groga 2014; e, Font Groga 2015.

In blue color, appear Jastrebac Mt. samples from 2023 (numbers 21 and 23) and, in red color, those from Jastrebac Mt. samples from previous years (numbers 18-20 and 22).
