# Supplementary material for: Temporal and habitat adaptations in Drosophila subobscura populations: changes in chromosomal inversions
Source: Genetica. 2025 Apr 25;153(1):16. doi: 10.1007/s10709-025-00232-9 (PMC12031780; doi:10.1007/s10709-025-00232-9)
Supplement: Supplementary file 2 — Supplementary Material 2. [file 10709_2025_232_MOESM2_ESM.docx]

**Supplementary Figure S2** Phylogenic tree using the O chromosomal inversions from Jastrebac Mt. and other Balkan and Font Groga (Barcelona) populations.


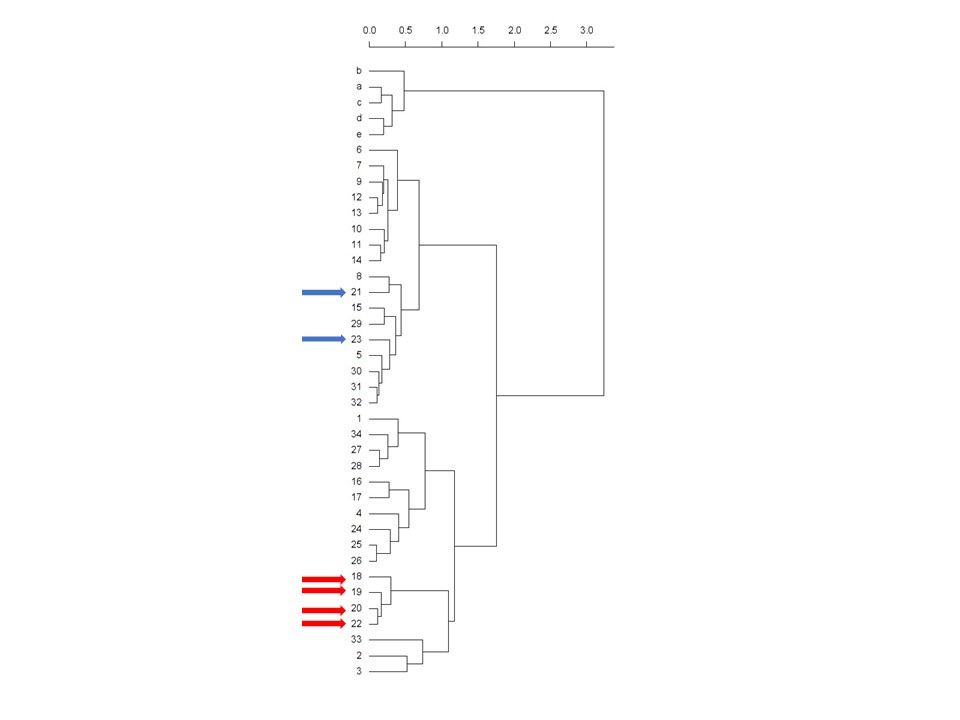


Numbers and letters indicate the same populations as in Supplementary Figure 1. Blue arrows indicate Jastrebac Mt. samples from 2023 (numbers 21 and 23) and red arrows those from Jastrebac Mt. samples from previous years (numbers 18-20 and 22).
