# Supplementary material for: Temporal and habitat adaptations in Drosophila subobscura populations: changes in chromosomal inversions
Source: Genetica. 2025 Apr 25;153(1):16. doi: 10.1007/s10709-025-00232-9 (PMC12031780; doi:10.1007/s10709-025-00232-9)

**Supplementary Figure S3** Changes over time (1991-2023) of the climatic variables (Tmean, Tmax, Tmin, difference between Tmax and T min, humidity and rainfall) in Jastrebac Mt.

**A.** Tmean
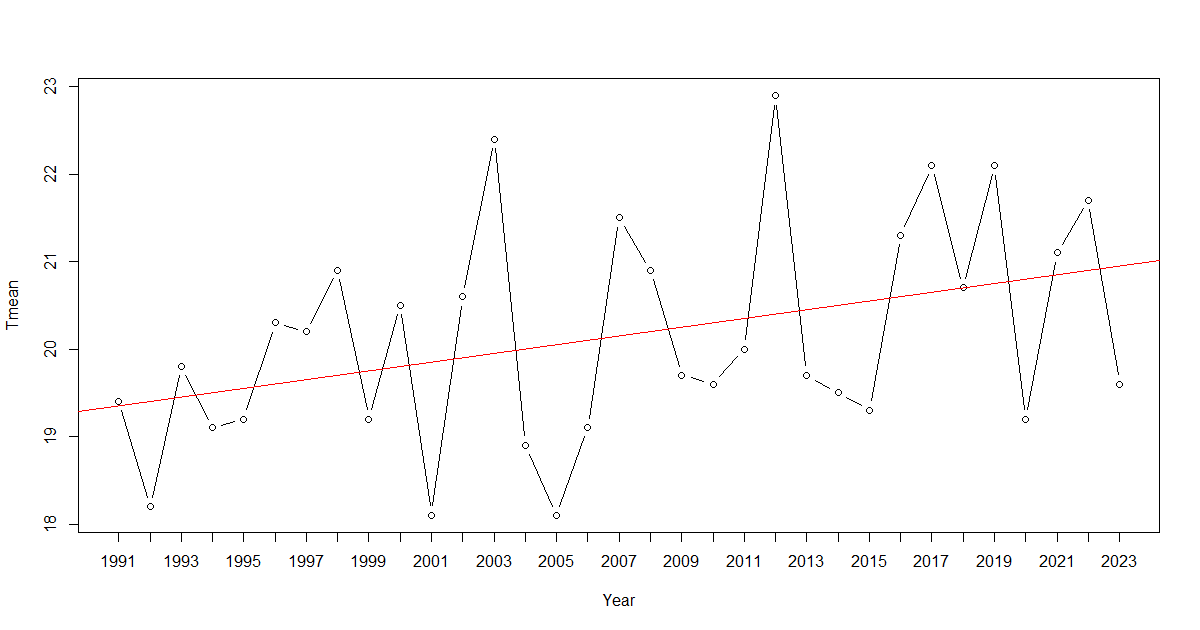


**B.** Tmax


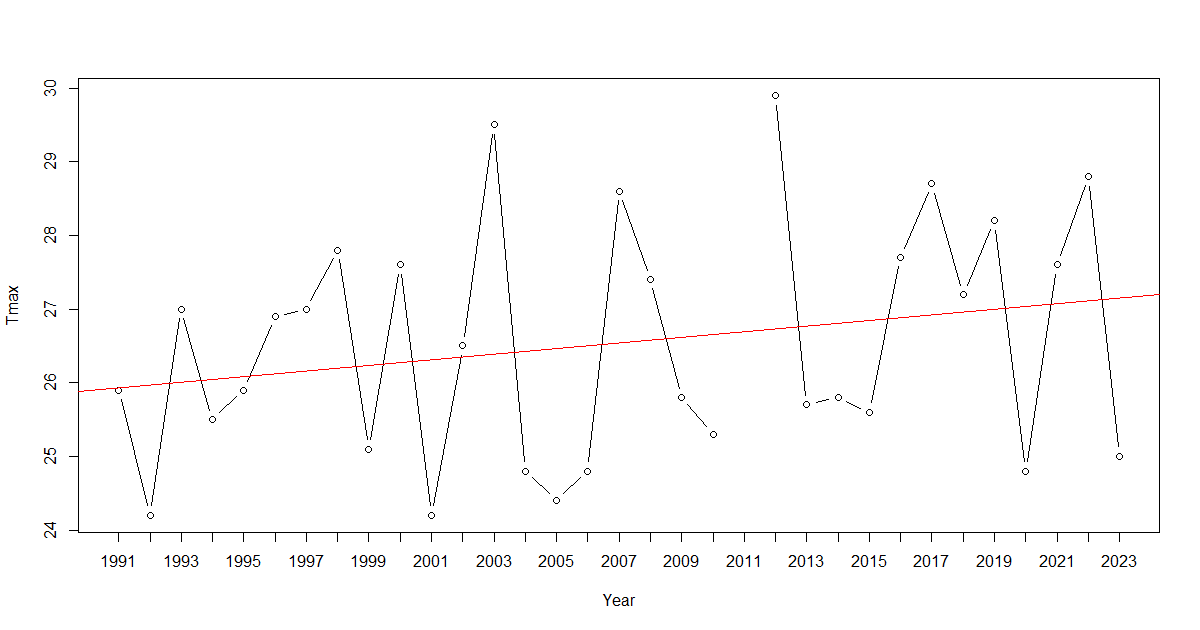


**C.** Tmin


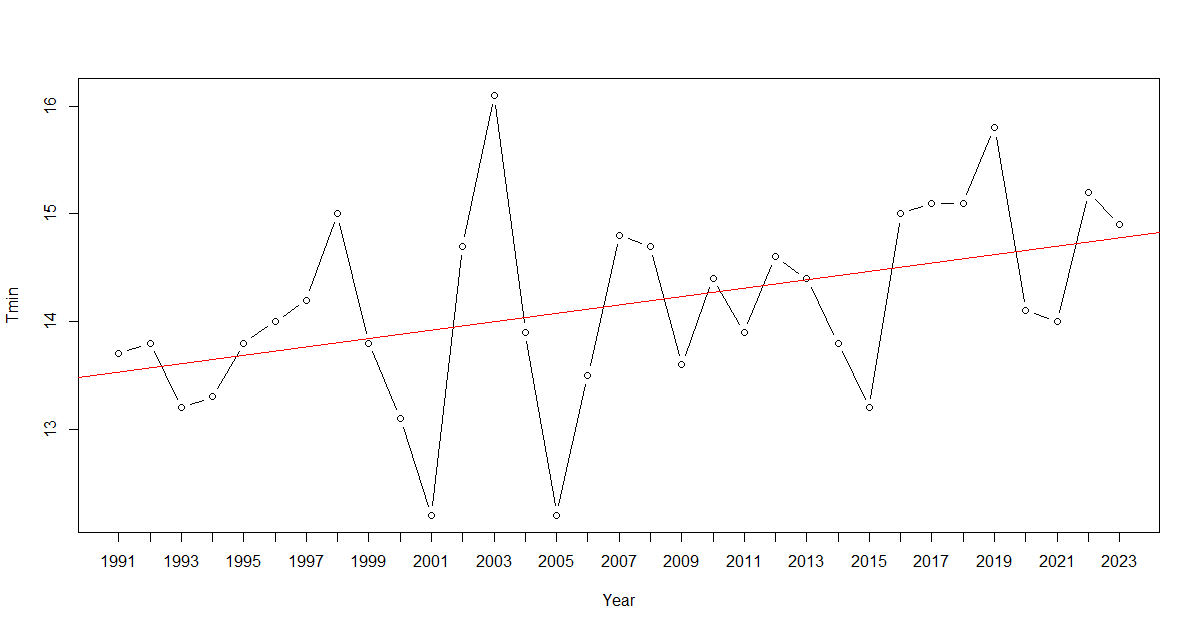


**D.** Difference between Tmax and Tmin


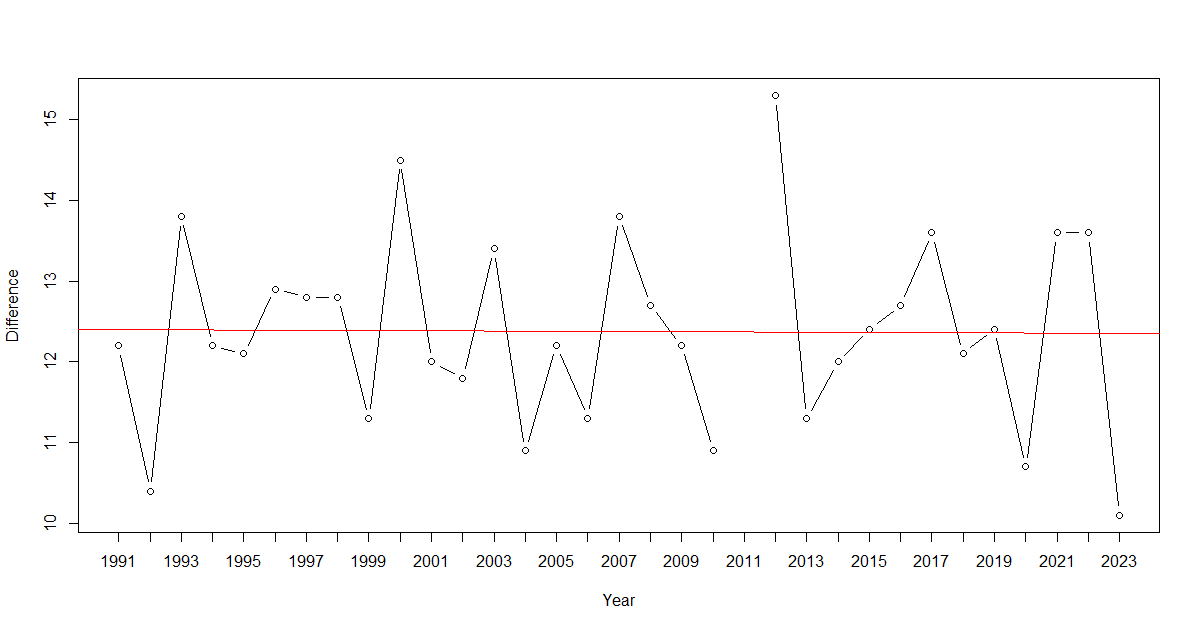


**E.** Humidity


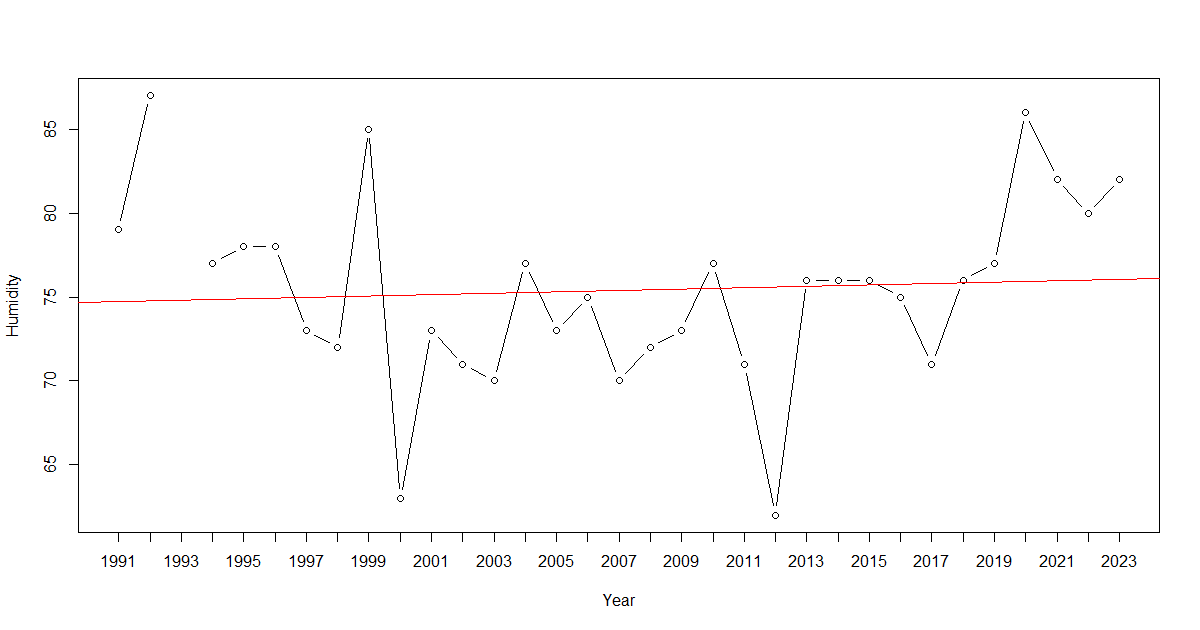


**F.** Rainfall


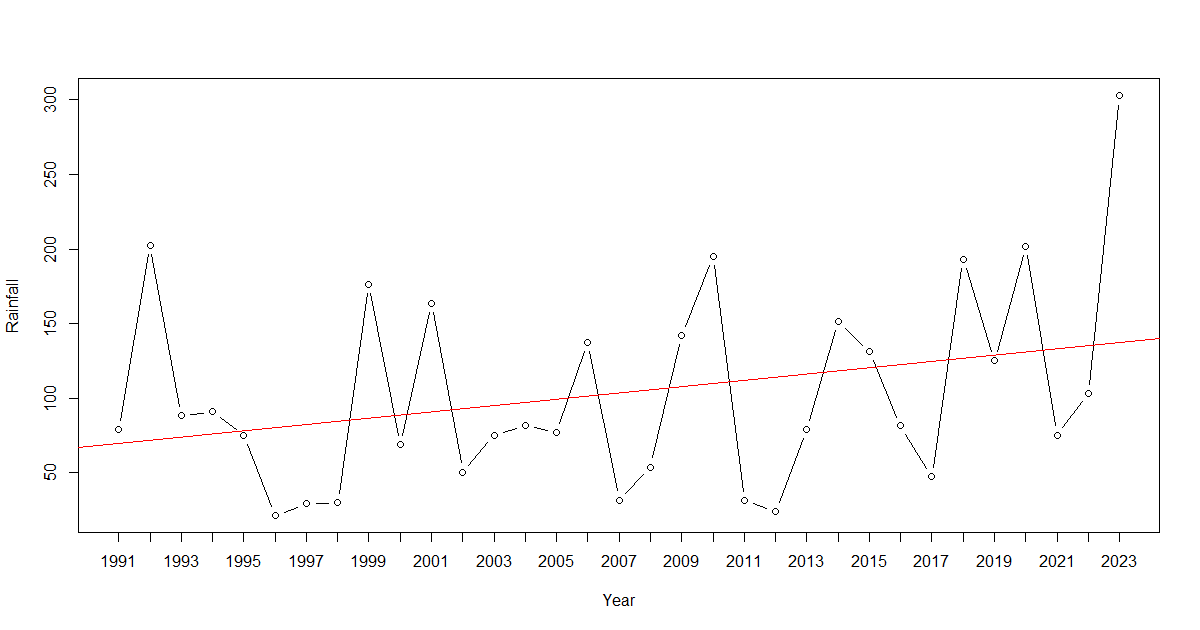

Supplement: Supplementary file 3 — Supplementary Material 3. [file 10709_2025_232_MOESM3_ESM.docx]
