# Supplementary material for: Temporal and habitat adaptations in Drosophila subobscura populations: changes in chromosomal inversions
Source: Genetica. 2025 Apr 25;153(1):16. doi: 10.1007/s10709-025-00232-9 (PMC12031780; doi:10.1007/s10709-025-00232-9)

**Supplementary Figure S4** Results of MDS analysis analyzing the relation between the inversion polymorphism of Serbian populations sampled in the month of June and climatic variables. Populations appear distributed in the plot according to their chromosomal inversion composition, and the effect of the corresponding variable is depicted as circles (a large circle corresponds to a large effect and a small one to a small effect).

The abbreviations for populations are: JB94, Jastrebac beech forest 1994; JB23, Jastrebac beech forest 2023; AP94, Apatin 1994; AP18, Apatin 2018; AV04, Avala 2004; AV11, Avala 2011; AV14, Avala 2014; AV15, Avala 2015; AV16, Avala 2016; AV17, Avala 2017; PE95, Petnica 1995; PE19, Petnica 2019; PE20, Petnica 2020; PE21, Petnica 2021; PE22, Petnica 2022; DJ01, Djerdap 2001; DJ02, Djerdap 2002; KA96, Kamariste 1996. The abbreviations for climatic variables are: Tmean (mean temperature), Tmax (maximum temperature), Tmin (minimum temperature), humidity and rainfall.

**A.** Tmean


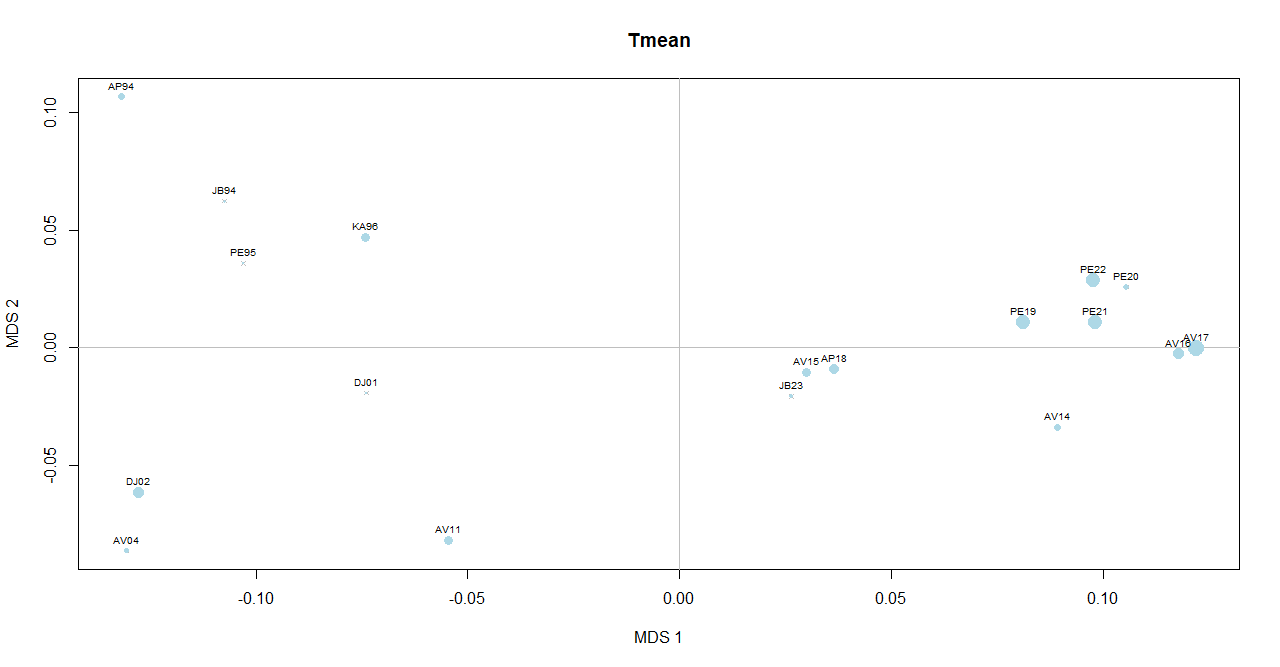


**B.** Tmax


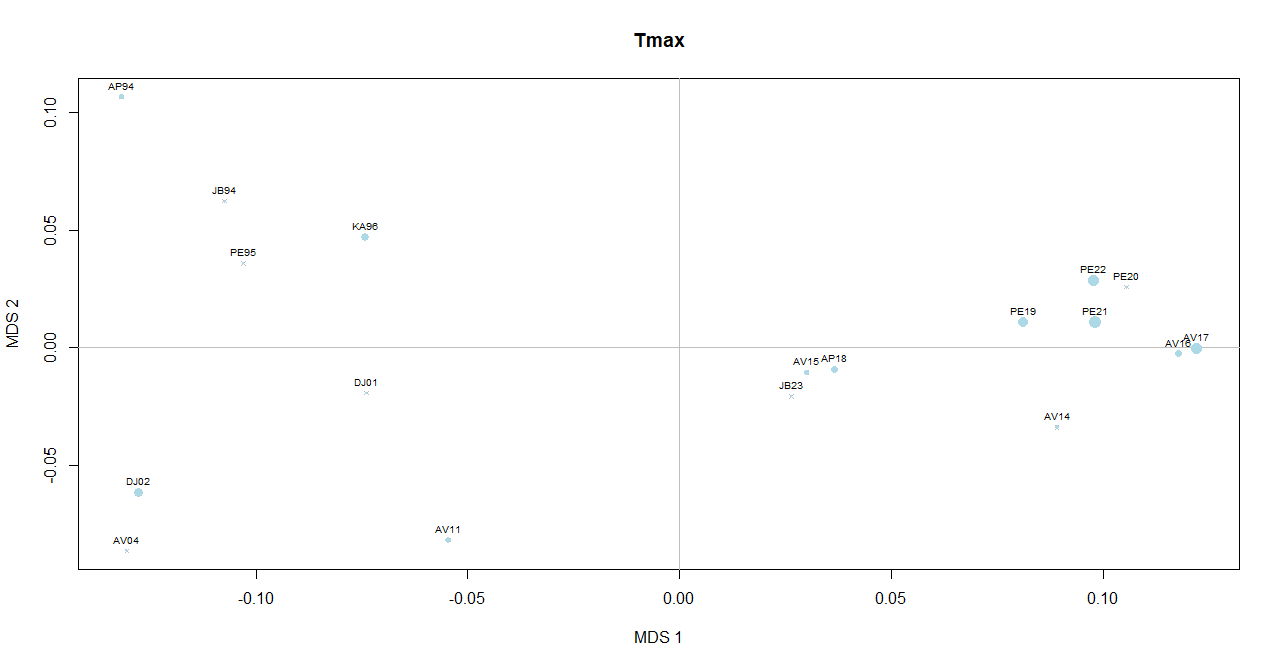


**C.** Tmin


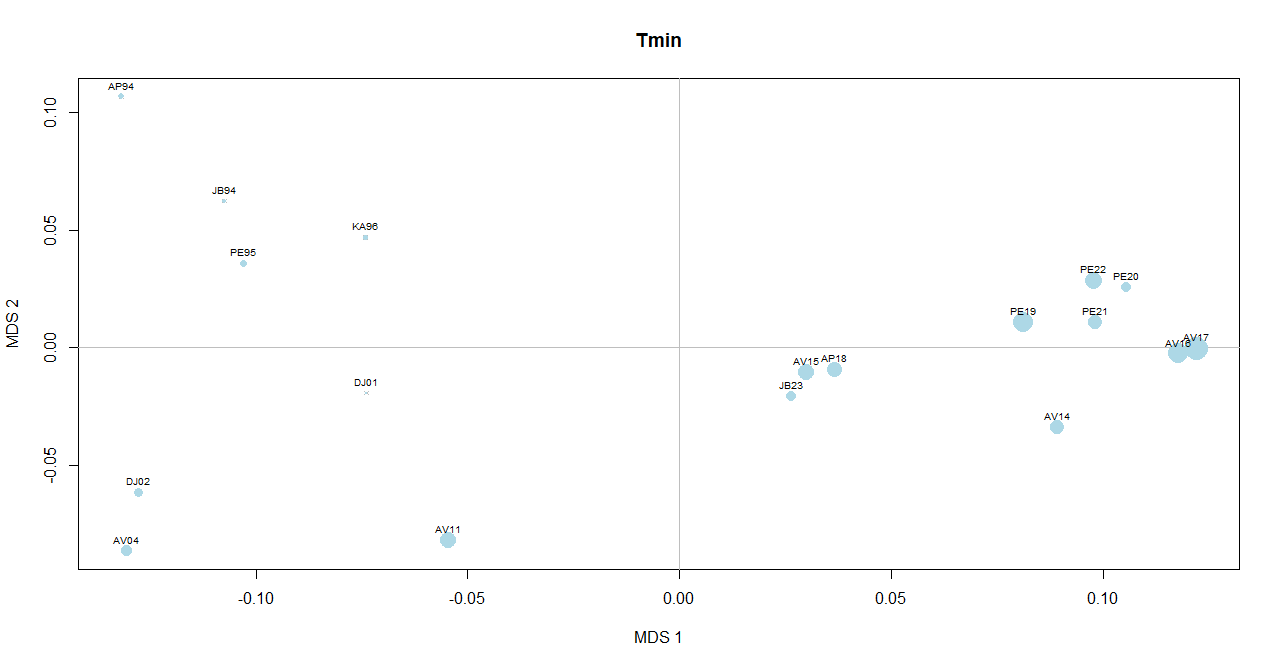


**D.** Humidity
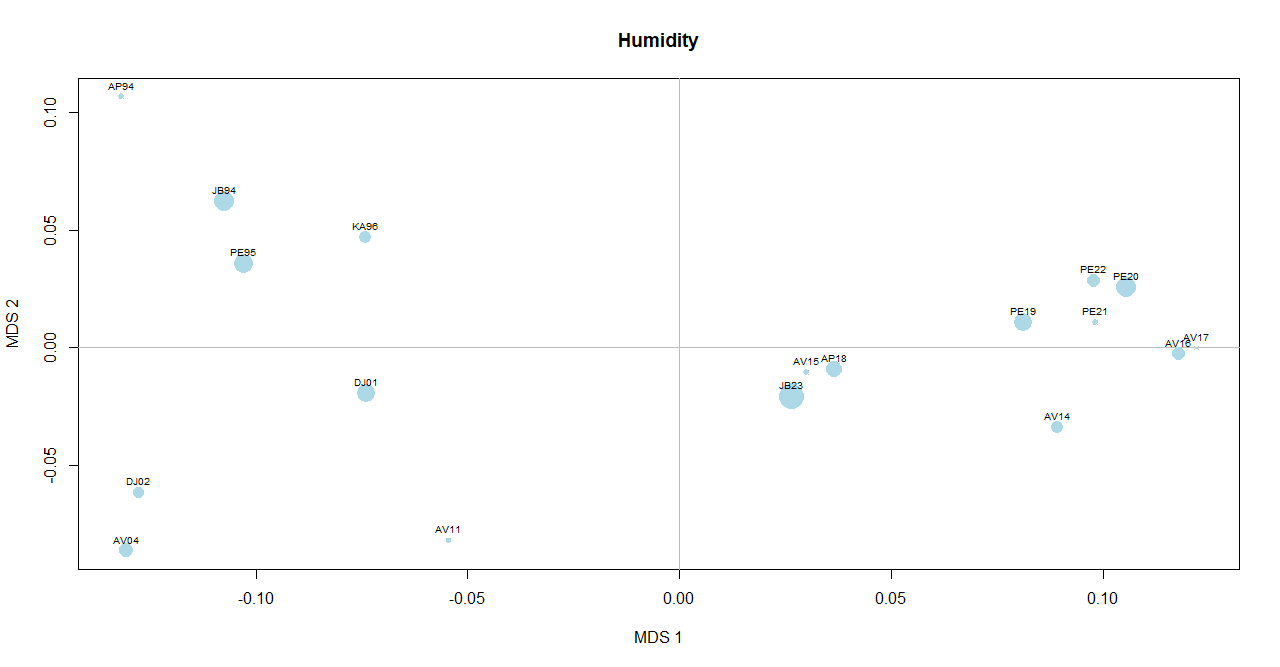


**E.** Rainfall


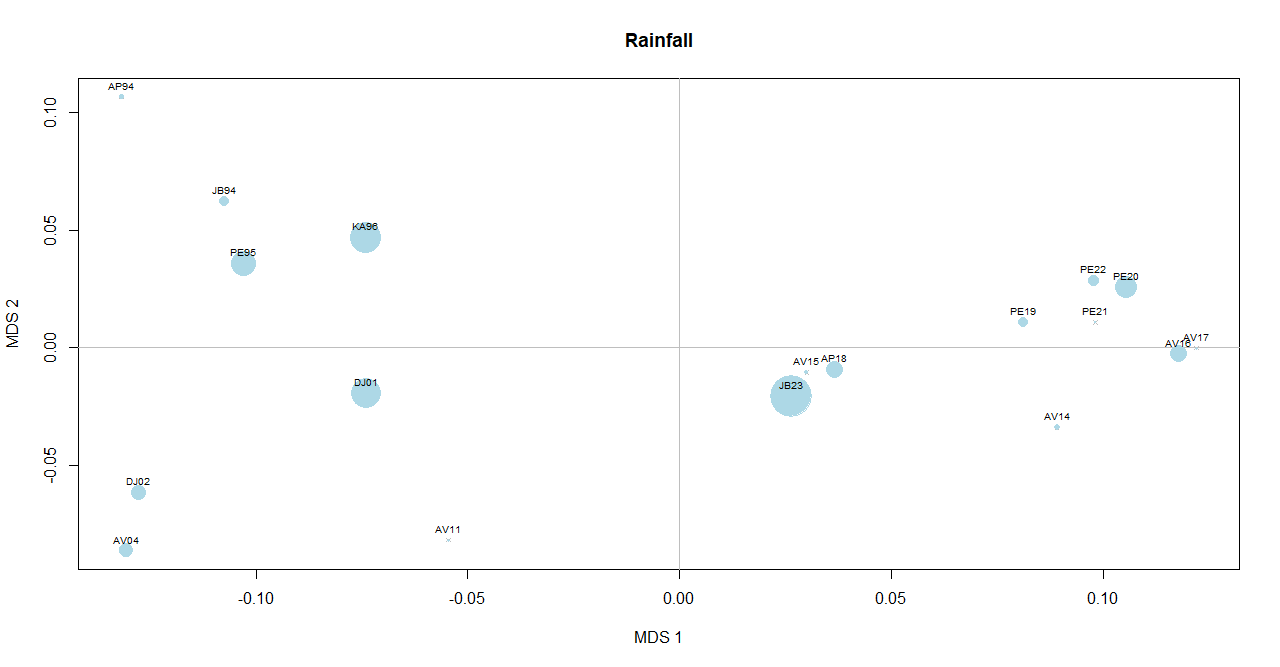

Supplement: Supplementary file 4 — Supplementary Material 4. [file 10709_2025_232_MOESM4_ESM.docx]
