# Supplementary material for: Temporal and habitat adaptations in Drosophila subobscura populations: changes in chromosomal inversions
Source: Genetica. 2025 Apr 25;153(1):16. doi: 10.1007/s10709-025-00232-9 (PMC12031780; doi:10.1007/s10709-025-00232-9)
Supplement: Supplementary file 5 — Supplementary Material 5. [file 10709_2025_232_MOESM5_ESM.docx]

**Supplementary Table S1** Frequencies of *D. subobscura* chromosomal arrangements from the beech forest of Jastrebac Mt. in June for different years (1990, 1993, 1994 and 2023). Data for years 1990, 1993 and 1994 are from Zivanovic et al. (1995).

|  |  | |  | |  | |  | |
| --- | --- | --- | --- | --- | --- | --- | --- | --- |
|  |  | |  | |  | |  | |
| Chrom. arrangements | June 1990 | | June 1993 | | June 1994 | | June 2023 | |
|  | n | % | n | % | n | % | n | % |
| A_st_ | 44 | 88.0 | 49 | 48.0 | 25 | 41.7 | 27 | 33.7 |
| A_1_ | 6 | 12.0 | 40 | 39.2 | 24 | 40.0 | 26 | 32.5 |
| A_2_ | 0 | 0 | 13 | 12.8 | 11 | 18.3 | 27 | 33.7 |
| Total | 50 |  | 102 |  | 60 |  | 80 |  |
| J_st_ | 21 | 21.0 | 51 | 25.0 | 15 | 12.5 | 35 | 21.8 |
| J_1_ | 78 | 78.0 | 153 | 75.0 | 105 | 87.5 | 123 | 76.8 |
| J_3+4_ | 1 | 1.0 | 0 | 0 | 0 | 0 | 2 | 1.2 |
| Total | 100 |  | 204 |  | 120 |  | 160 |  |
| U_st_ | 12 | 12.0 | 22 | 10.8 | 11 | 9.2 | 11 | 6.8 |
| U_1_ | 4 | 4.0 | 3 | 1.5 | 1 | 0.8 | 2 | 1.2 |
| U_1+2_ | 69 | 69.0 | 94 | 46.1 | 71 | 59.2 | 84 | 52.5 |
| U_1+2+3_ | 1 | 1.0 | 1 | 0.5 | 0 | 0 | 3 | 1.8 |
| U_1+2+6_ | 14 | 14.0 | 84 | 41.2 | 37 | 30.8 | 52 | 32.5 |
| U_1+8+2_ | 0 | 0 | 0 | 0 | 0 | 0 | 8 | 5.0 |
| Total | 100 |  | 204 |  | 120 |  | 160 |  |
| E_st_ | 38 | 38.0 | 65 | 31.8 | 31 | 25.8 | 46 | 28.7 |
| E_1+2_ | 3 | 3.0 | 2 | 1.0 | 6 | 5.0 | 8 | 5.0 |
| E_1+2+9_ | 37 | 37.0 | 85 | 41.7 | 50 | 41.7 | 64 | 40.0 |
| E_1+2+9+12_ | 0 | 0 | 0 | 0 | 0 | 0 | 7 | 4.3 |
| E_8_ | 22 | 22.0 | 52 | 25.5 | 33 | 27.5 | 35 | 21.8 |
| Total | 100 |  | 204 |  | 120 |  | 160 |  |
| O_st_ | 27 | 27.0 | 54 | 26.5 | 21 | 17.5 | 27 | 16.8 |
| O_15_ | 2 | 2.0 | 0 | 0 | 0 | 0 | 0 | 0 |
| O_3+4_ | 42 | 42.0 | 103 | 50.5 | 63 | 52.5 | 90 | 56.2 |
| O_3+4+1_ | 24 | 24.0 | 40 | 19.6 | 30 | 25.0 | 24 | 15.0 |
| O_3+4+2_ | 2 | 2.0 | 4 | 2.0 | 5 | 4.2 | 0 | 0 |
| O_3+4+6_ | 0 | 0 | 0 | 0 | 0 | 0 | 5 | 3.1 |
| O_3+4+7_ | 0 | 0 | 2 | 1.0 | 1 | 0.8 | 3 | 1.8 |
| O_3+4+8_ | 3 | 3.0 | 1 | 0.5 | 0 | 0 | 4 | 2.5 |
| O_3+4+17_ | 0 | 0 | 0 | 0 | 0 | 0 | 1 | 0.6 |
| O_3+4+22_ | 0 | 0 | 0 | 0 | 0 | 0 | 6 | 3.7 |
| Total | 100 |  | 204 |  | 120 |  | 160 |  |
| *CTI* | 0.262 |  | 0.270 |  | 0.444 |  | 0.430 |  |
