# Supplementary material for: Temporal and habitat adaptations in Drosophila subobscura populations: changes in chromosomal inversions
Source: Genetica. 2025 Apr 25;153(1):16. doi: 10.1007/s10709-025-00232-9 (PMC12031780; doi:10.1007/s10709-025-00232-9)
Supplement: Supplementary file 6 — Supplementary Material 6. [file 10709_2025_232_MOESM6_ESM.docx]

**Supplementary Table S2** Frequencies of *D. subobscura* chromosomal arrangements from the oak forest of Jastrebac Mt. in June for 1990 (Zivanovic et al. 1995) and 2023.

|  | | | | |
| --- | --- | --- | --- | --- |
|  | | | | |
| Chrom. arrangements | June 1990 | | June 2023 | |
|  | n | % | n | % |
| A_st_ | 29 | 58.0 | 19 | 27.1 |
| A_1_ | 13 | 26.0 | 42 | 60.0 |
| A_2_ | 8 | 16.0 | 9 | 12.8 |
| Total | 50 |  | 70 |  |
| J_st_ | 31 | 31.0 | 31 | 22.1 |
| J_1_ | 69 | 69.0 | 107 | 76.4 |
| J_3+4_ | 0 | 0 | 2 | 1.4 |
| Total | 100 |  | 140 |  |
| U_st_ | 4 | 4.0 | 16 | 11.4 |
| U_1_ | 0 | 0 | 2 | 1.4 |
| U_1+2_ | 80 | 80.0 | 74 | 52.8 |
| U_1+2+3_ | 0 | 0 | 3 | 2.1 |
| U_1+2+6_ | 16 | 16.0 | 41 | 29.3 |
| U_1+8+2_ | 0 | 0 | 4 | 2.8 |
| Total | 100 |  | 140 |  |
| E_st_ | 24 | 24.0 | 48 | 34.3 |
| E_1+2_ | 5 | 5.0 | 6 | 4.3 |
| E_1+2+9_ | 45 | 45.0 | 43 | 30.7 |
| E_1+2+9+12_ | 0 | 0 | 13 | 9.3 |
| E_8_ | 26 | 26.0 | 30 | 21.4 |
| Total | 100 |  | 140 |  |
| O_st_ | 21 | 21.0 | 33 | 23.6 |
| O_6_ | 0 | 0 | 1 | 0.7 |
| O_3+4_ | 50 | 50.0 | 55 | 39.3 |
| O_3+4+1_ | 27 | 27.0 | 14 | 10.0 |
| O_3+4+2_ | 2 | 2.0 | 0 | 0 |
| O_3+4+6_ | 0 | 0 | 4 | 2.8 |
| O_3+4+7_ | 0 | 0 | 4 | 2.8 |
| O_3+4+8_ | 0 | 0 | 11 | 7.8 |
| O_3+4+22_ | 0 | 0 | 18 | 12.8 |
| Total | 100 |  | 140 |  |
| *CTI* | 0.392 |  | 0.272 |  |
