# Supplementary material for: Temporal and habitat adaptations in Drosophila subobscura populations: changes in chromosomal inversions
Source: Genetica. 2025 Apr 25;153(1):16. doi: 10.1007/s10709-025-00232-9 (PMC12031780; doi:10.1007/s10709-025-00232-9)
Supplement: Supplementary file 8 — Supplementary Material 8 [file 10709_2025_232_MOESM8_ESM.docx]

**Supplementary Table S4.** Comparisons between years and habitat (1990 beech, 2023 beech, 1990 oak and 2023 oak) for all chromosomes (A, J, U, E and O), considering the thermal adaptations composition of inversions (‘cold’, ‘warm’ and ‘non-thermal’ adapted) from Jastrebac Mt. The values of *P* and adjusted *P* are presented. Significant values are in bold.

| **COLD INVERSIONS** | *P* | *P (adjusted)* |
| --- | --- | --- |
| A chromosome |  |  |
| 1990B *vs*. 2023B | 0.6315 | 0.9851 |
| 1990O *vs*. 2023O | 0.7125 | 0.9851 |
| 1990B *vs*. 1990O | 1.0000 | 1.0000 |
| 2023B *vs*. 2023O | 0.8209 | 0.9851 |
| 1990B *vs*. 2023O | 0.8157 | 0.9851 |
| 1990O *vs*. 2023B | 0.5291 | 0.9851 |
|  |  |  |
| J chromosome |  |  |
| 1990B *vs*. 2023B | 0.1841 | 0.3682 |
| 1990O *vs*. 2023O | 0.0594 | 0.1781 |
| 1990B *vs*. 1990O | **0.0293** | 0.1757 |
| 2023B *vs*. 2023O | 0.3434 | 0.4121 |
| 1990B *vs*. 2023O | 0.6495 | 0.6495 |
| 1990O *vs*. 2023B | 0.3232 | 0.4121 |
|  |  |  |
| U chromosome |  |  |
| 1990B *vs*. 2023B | 0.6654 | 0.7985 |
| 1990O *vs*. 2023O | 0.0960 | 0.3653 |
| 1990B *vs*. 1990O | 0.1218 | 0.3653 |
| 2023B *vs*. 2023O | 0.5494 | 0.7985 |
| 1990B *vs*. 2023O | 1.0000 | 1.0000 |
| 1990O *vs*. 2023B | 0.2888 | 0.5776 |
|  |  |  |
| E chromosome |  |  |
| 1990B *vs*. 2023B | 0.8988 | 1.0000 |
| 1990O *vs*. 2023O | 0.2722 | 0.5443 |
| 1990B *vs*. 1990O | 0.2497 | 0.5443 |
| 2023B *vs*. 2023O | 0.8107 | 1.0000 |
| 1990B *vs*. 2023O | 1.0000 | 1.0000 |
| 1990O *vs*. 2023B | 0.1679 | 0.5443 |
|  |  |  |
| O chromosome |  |  |
| 1990B *vs*. 2023B | 0.5533 | 1.0000 |
| 1990O *vs*. 2023O | 1.0000 | 1.0000 |
| 1990B *vs*. 1990O | 0.8738 | 1.0000 |
| 2023B *vs*. 2023O | 0.6736 | 1.0000 |
| 1990B *vs*. 2023O | 0.8865 | 1.0000 |
| 1990O *vs*. 2023B | 0.7507 | 1.0000 |
|  |  |  |

| **WARM INVESIONS** | *P* | *P (adjusted)* |
| --- | --- | --- |
| A chromosome |  |  |
| 1990B vs. 2023B | **0.0000** | **0.0000** |
| 1990O vs. 2023O | 1.0000 | 1.0000 |
| 1990B vs. 1990O | **0.0079** | **0.0158** |
| 2023B vs. 2023O | **0.0249** | **0.0373** |
| 1990B vs. 2023O | **0.0062** | **0.0158** |
| 1990O vs. 2023B | 0.0500 | 0.0600 |
|  |  |  |
| J chromosome |  |  |
| 1990B *vs*. 2023B | 0.5434 | 0.6521 |
| 1990O *vs*. 2023O | **0.0393** | 0.2360 |
| 1990B *vs*. 1990O | 0.1214 | 0.3641 |
| 2023B *vs*. 2023O | 0.2651 | 0.4490 |
| 1990B *vs*. 2023O | 0.7198 | 0.7198 |
| 1990O *vs*. 2023B | 0.2993 | 0.4490 |
|  |  |  |
| U chromosome |  |  |
| 1990B *vs*. 2023B | **0.0012** | **0.0036** |
| 1990O *vs*. 2023O | 0.1650 | 0.2474 |
| 1990B *vs*. 1990O | 0.7720 | 0.7720 |
| 2023B *vs*. 2023O | **0.0213** | **0.0427** |
| 1990B *vs*. 2023O | 0.3366 | 0.4039 |
| 1990O *vs*. 2023B | **0.0002** | **0.0011** |
|  |  |  |
| E chromosome |  |  |
| 1990B *vs*. 2023B | 0.5874 | 0.9219 |
| 1990O *vs*. 2023O | 0.8273 | 0.9219 |
| 1990B *vs*. 1990O | 0.7185 | 0.9219 |
| 2023B *vs*. 2023O | 0.9219 | 0.9219 |
| 1990B *vs*. 2023O | 0.4941 | 0.9219 |
| 1990O *vs*. 2023B | 0.9177 | 0.9219 |
|  |  |  |
| O chromosome |  |  |
| 1990B *vs*. 2023B | 1.0000 | 1.0000 |
| 1990O *vs*. 2023O | 0.3540 | 0.8874 |
| 1990B *vs*. 1990O | 1.0000 | 1.0000 |
| 2023B *vs*. 2023O | 0.3593 | 0.8874 |
| 1990B vs. 2023O | 0.4437 | 0.8874 |
| 1990O vs. 2023B | 1.0000 | 1.0000 |

| **NON-THERMAL INVERSIONS** | *P* | *P (adjusted)* |
| --- | --- | --- |
|  |  |  |
| J chromosome |  |  |
| 1990B *vs*. 2023B | 1.0000 | 1.0000 |
| 1990O *vs*. 2023O | 1.0000 | 1.0000 |
| 1990B *vs*. 1990O | 1.0000 | 1.0000 |
| 2023B *vs*. 2023O | 1.0000 | 1.0000 |
| 1990B *vs*. 2023O | 1.0000 | 1.0000 |
| 1990O *vs*. 2023B | 1.0000 | 1.0000 |
|  |  |  |
| U chromosome |  |  |
| 1990B *vs*. 2023B | 0.1794 | 0.4439 |
| 1990O *vs*. 2023O | 0.2960 | 0.4439 |
| 1990B *vs*. 1990O | 0.6736 | 0.8083 |
| 2023B *vs*. 2023O | 0.2345 | 0.4439 |
| 1990B *vs*. 2023O | 0.8615 | 0.8615 |
| 1990O *vs*. 2023B | **0.0170** | 0.2502 |
|  |  |  |
| E chromosome |  |  |
| 1990B *vs*. 2023B | 0.0835 | 0.1252 |
| 1990O *vs*. 2023O | **0.0005** | **0.0028** |
| 1990B *vs*. 1990O | 0.3074 | 0.3689 |
| 2023B *vs*. 2023O | 0.6789 | 0.6789 |
| 1990B *vs*. 2023O | **0.0346** | 0.0691 |
| 1990O *vs*. 2023B | **0.0012** | **0.0035** |
|  |  |  |
| O chromosome |  |  |
| 1990B *vs*. 2023B | 0.5928 | 0.6775 |
| 1990O *vs*. 2023O | **0.0015** | **0.0091** |
| 1990B *vs*. 1990O | 0.6775 | 0.6775 |
| 2023B *vs*. 2023O | **0.0256** | 0.0512 |
| 1990B *vs*. 2023O | **0.0170** | 0.0509 |
| 1990O *vs*. 2023B | 0.1565 | 0.2348 |

B and O stand for ‘beech’ and ‘oak’, respectively.
